# Characterisation of Temnocephalidae flatworms in common Australian freshwater prawn, *Macrobrachium australiense*

**DOI:** 10.1038/s41598-022-05123-z

**Published:** 2022-01-26

**Authors:** Shokoofeh Shamsi, Lachlan Sibraa, Xiaocheng Zhu, Diane P. Barton

**Affiliations:** 1 School of Agriculture, Environment and Veterinary Sciences, Charles Sturt University, Wagga Wagga, 2678 Australia; 2 Graham Centre for Agricultural Innovation, Charles Sturt University, Wagga Wagga, 2678 Australia; 3 NSW Department of Primary Industries, Wagga Wagga Agricultural Institute, Wagga Wagga, NSW 2650 Australia

**Keywords:** Microbiology, Parasitology

## Abstract

*Macrobrachium australiense*, is one of Australia’s most widespread freshwater invertebrates. Although a significant amount of research has been conducted to understand the diversity of crustacean species in Australia, there has been considerably less effort focused on their Temnocephalidae symbionts. The present study aims to identify Temnocephalidae species found in *M. australiense*, along with determining their impacts on the fitness of their hosts. A total of 54 M*. australiense* (common Australian river prawn) were examined for evidence of infection with Temnocephalidae species, of which 96.3% showed at least one sign of infection with Temnocephalidae. Due to damage and immaturity of the worms collected from*,* they have been referred to as Temnocephalidae sp. based on the presence of tentacles on the anterior margin of the body, and pedunculate sucker located dorsally on the ventral surface. Possible mechanical damage to gill lamellae resulting from either egg deposition or autolysis is evident. In the phylogenetic tree built based on sequences of the 28S rRNA gene, specimens in the present study grouped separately from other Temnocephalidae species reported from Australia.

## Introduction

The freshwater palaemonid shrimp, *Macrobrachium australiense* Holthuis, 1950, is one of Australia’s most widespread freshwater invertebrates throughout eastern Australian freshwater catchments^1^. *Macrobrachium australiense* is well adapted to freshwater environments and is one of the few *Macrobrachium* spp. that is able to complete their entire life cycle in freshwater^2^. Temnocephalans are Rhabditophoran ectosymbionts commonly associated with freshwater crustaceans, with eastern Australia being recognised as a hot-spot for Temnocephalan diversity^3^. Although a significant amount of research has been conducted to understand the diversity of crustacean species in Australia, there has been considerably less effort focused on their Temnocephalidae symbionts. It is likely that the diversity of both Australian crustacean and Temnocephalidae taxa is incomplete. The association between Australian temnocephalans and their hosts is ancient, with a shared history being evident based on phylogenetics^4^. However, while the relationship between these species is generally believed to be commensal in nature, with many species surviving and reproducing on the carapace of the host and feeding on debris present within their immediate vicinity^5^, there are a number of Temnocephalidae species that are found associated within the gill chamber of the host^6^, and to date there have been no studies that explore the impact of these species on their hosts fitness and respiratory ability. However, it is known that parasitic platyhelminths (such as Monogenea) infecting respiratory organs of aquatic animals may cause extensive damages on their hosts^7,8^. This is a cause for concern as according to the IUCN Red List Assessments, approximately half of all Australian crayfish/freshwater prawns are currently considered threatened, and due to the close relationship shared by their Temnocephalid ectosymbionts, it is possible that many Temnocephalid species are at risk of coextinction as well^4^. The present study aims to identify Temnocephalidae species found in *M. australiense*, along with determining their impacts on the fitness of their hosts.

## Material and methods

### Sample collection

**Figure 1:**
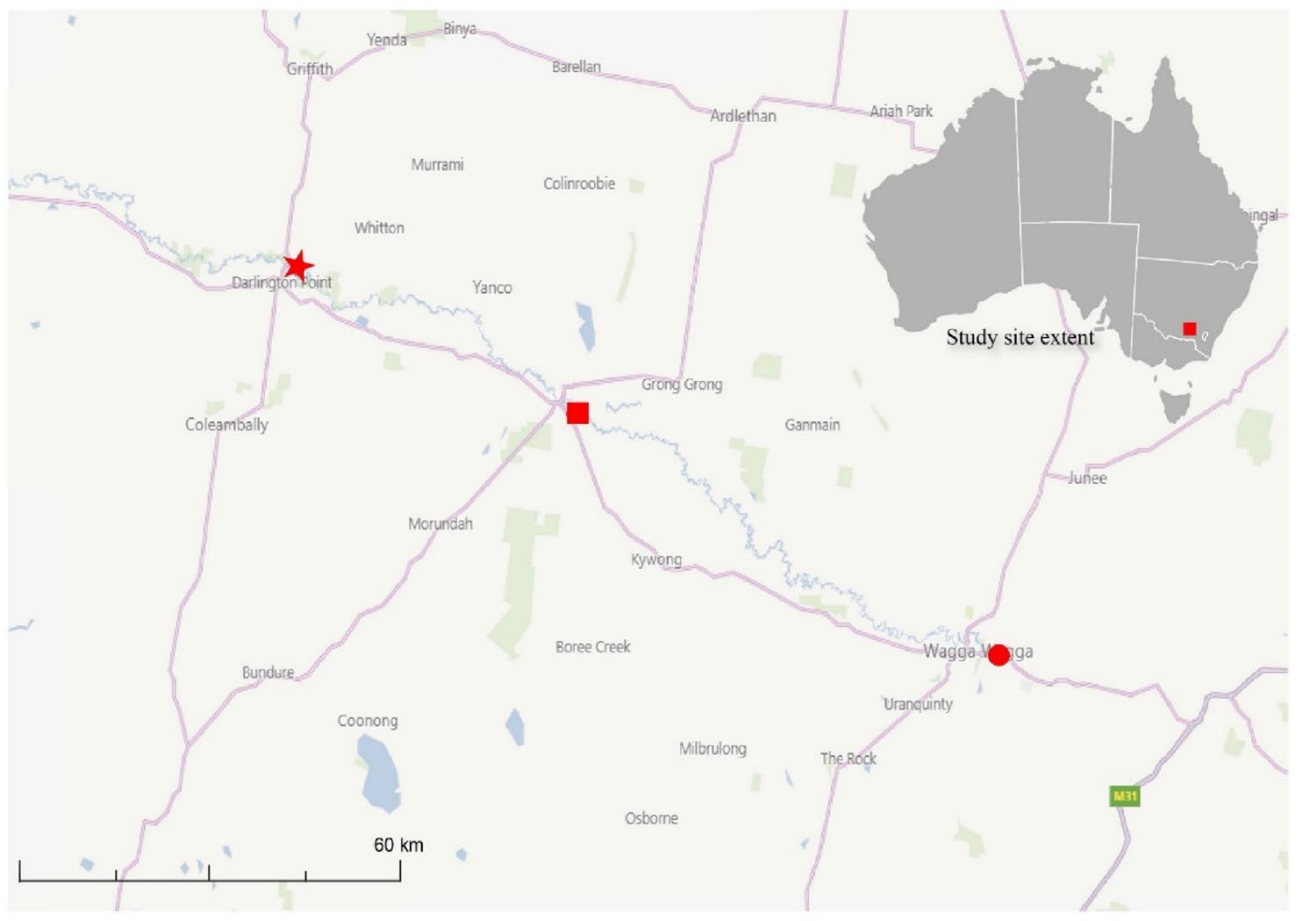
Star represents the Mantangery Lagoon sampling location (34° 35′ 30.41″ and 146° 4′ 12.76″) in the present study. Map was created by ArcGIS Pro (version 2.8.x -; ESRI (https://www.esri.com/en-us/home)).

*Macrobrachium australiense* specimens were sampled from Mantangery Lagoon, Darlington Point, New South Wales (Fig. 1). Individual crustacean specimens were measured and placed in a petri dish containing a small amount of fresh water, where they were examined using a dissecting microscope to detect the presence of temnocephalans. The entire external surface of each crustacean was examined for the presence of both adult temnocephalans and temnocephalan eggs. Forceps were used to hold the crustaceans in place, while a teasing needle was used to move the maxillipeds, pereiopods, pleopods, and uropods so that each could be examined individually. The carapace was then removed using fine forceps to examine the branchial chamber, gills, and branchial membranes for the presence of adult temnocephalans or eggs. All tissues that contained eggs were dissected out using fine forceps and placed in Eppendorf tubes containing 70% ethanol for future molecular analysis. Some samples were placed in formalin for future histopathology examination. All tubes and slides were labelled with the identification number of the crustacean being examined at the time. Following dissection, each crustacean was removed from the petri dish and the water remaining in the dish was examined for any adults or eggs that were displaced during dissection. All remaining adult temnocephalans and eggs were collected with a transfer pipette and placed in 70% ethanol for preservation. The petri dish and transfer pipettes were rinsed and cleaned with fresh water between each examination of crustaceans. Following examination of all *M. australiense* specimens, the bag was rinsed with water and the washing examined for any temnocephalans that may have been dislodged from the prawns during transport.

Subsequently, adult temnocephalans were utilised for both morphological and molecular analysis. The Eppendorf tubes containing the collected specimens were emptied one at a time into a watch glass and examined under a Leica EZ4 dissecting microscope (Leica Microsystems). Well-preserved adults and tissues infested with eggs were collected with the bristles of a fine paint brush or fine forceps and mounted on slides with glycerine jelly. Damaged adults were dissected with the use of fine forceps and a scalpel, with the anterior portions being placed into separate PCR tubes for future analysis, and posterior portions being mounted on slides with lactophenol for visualisation of the cirrus and vagina for morphological identification. The forceps and scalpel were washed with fresh water between each dissection to remove any genetic material that may have remained on the equipment. The watch glass was emptied and rinsed with fresh water in between each sample examination.

### Specimens examination and characterisation

Identification of Temnocephalidae species were according to Sewell, et al.^9^ and Sewell and Cannon^10^. Posterior sections mounted with lactophenol were examined under a compound light microscope to view the reproductive structures and were then compared to formal descriptions to identify the specimens to a species level.

DNA extraction of anterior portions of temnocephalans was achieved using DNeasy Blood and Tissue Kits (Qiagen, Australia), according to the modified version of the manufacturer’s instructions^11^. The 28S rRNA gene region was amplified using the primer pair EC-D2 (5’-CCTTGGTCCGTGTTTCAAGACGGG-3’) and LSU4 (5’-TAGGTCGACCCGCTGAAYTTAAGCA-3’)^12^ using the same reagents published previously^13^ . PCR conditions were set as follows: 95° C initial denature for 2.5 min, followed by four cycles of touchdown PCR 30 s at 95° C, 30 s at 49° C minus 2° C every two cycles and 30 s at 72° C; then, 34 regular cycles of 30 s at 95° C, 30 s at 45° C, and 30 s at 72 °C. The cycle is ended with 10 min final extension at 72° C. PCR amplicons were bidirectional sequenced using the same PCR primers by Australian Genome Research Facility (Queensland). The 28S rRNA sequences were either generated in the current study or were obtained from GenBank (Table 1).

**Table 1:** Details of sequences used to build phylogenetic tree in the present study.

Species name	Accession no	Host species	Locality	Publication
*Temnosewellia dendyi*	KX095258	*Cherax destructor*	QLD	Cuthill et al.^4^
*Temnosewellia minor*	KX095257, AY157164	*Cherax destructor*	QLD, NSW	Cuthill et al.^4^; Lockyer et al.^24^
*Temnosewellia acicularis*	KX095259	*Euastcus bidawalus*	VIC	Cuthill et al.^4^
*Temnosewellia alba*	KX095260	*Euastacus c.f. balanensis*	QLD	Cuthill et al.^4^
*Temnosewellia albata*	KX095262	*Euastacus robertsi*	QLD	Cuthill et al.^4^
*Temnosewellia aphyodes*	KX095263	*Euastacus fleckeri*	QLD	Cuthill et al.^4^
*Temnosewellia apiculus*	KX095264	*Euastacus kershawi*	VIC	Cuthill et al.^4^
*Temnosewellia arga*	KX095265	*Euastacus yigara*	QLD	Cuthill et al.^4^
*Temnosewellia aspinosa*	KX095266	*Euastacus valentulus*	QLD	Cuthill et al.^4^
*Temnosewellia bacrioniculus*	KX095268	*Euastacus neohirsutus*	NSW	Cuthill et al.^4^
*Temnosewellia batiola*	KX095271	*Euastacus hystricosus*	QLD	Cuthill et al.^4^
*Temnosewellia comythus*	KX095273	*Euastacus spinichelatus*	NSW	Cuthill et al.^4^
*Temnosewellia coughrani*	KX095276	*Euastacus sulcatus*	NSW	Cuthill et al.^4^
*Temnosewellia fasciata*	KX095279	*Euastacus spinifer*	NSW	Cuthill et al.^4^
*Temnosewellia fax*	KX095281	*Euastacus yanga*	NSW	Cuthill et al.^4^
*Temnosewellia flammula*	KX095283	*Euastacus neohirsutus*	NSW	Cuthill et al.^4^
*Temnosewellia gingrina*	KX095284	*Euastacus sulcatus*	NSW	Cuthill et al.^4^
*Temnosewellia gracilis*	KX095288	*Euastacus guwinus*	NSW	Cuthill et al.^4^
*Temnosewellia keras*	KX095289	*Euastacus yarraensis*	VIC	Cuthill et al.^4^
*Temnosewellia muscalingulata*	KX095290	*Euastacus rieki*	NSW	Cuthill et al.^4^
*Temnosewellia maculate*	KX095292	*Euastacus bispinosus*	VIC	Cuthill et al.^4^
*Temnosewellia minima*	KX095293	*Euastacus sulcatus*	QLD	Cuthill et al.^4^
*Temnosewellia unguiculus*	KX095294	*Euastacus claytoni*	NSW	Cuthill et al.^4^
*Temnohaswellia alpine*	KX095295	*Euastacus rieki*	NSW	Cuthill et al.^4^
*Temnohaswellia Capricornia*	KX095296	*Euastacus monteithorum*	QLD	Cuthill et al.^4^
*Temnohaswellia comes*	KX095297–KX095300, KX095302–KX095305	*Euastacus gumar, E. mirangudjin, E. neohirsutus, E. spinichelatus, E. spinifer, E. sulcatus, E. suttoni*	NSW	Cuthill et al.^4^
*Temnohaswellia crotalum*	KX095307, KX095308	*Euastacus yarraensis, E. woiwuru*	VIC	Cuthill et al.^4^
*Temnohaswellia munifica*	KX095309	*Euastacus hystricosus*	QLD	Cuthill et al.^4^
*Temnohaswellia pearsoni*	KX095310	*Euastacus eungella*	QLD	Cuthill et al.^4^
*Temnohaswellia simulator*	KX095311–KX095316	*Euastacus gumar, E. spinichelatus, E. dangadi, E. neohirsutus, E. sulcatus*	NSW, QLD	Cuthill et al.^4^
*Temnohaswellia subulata*	KX095317	*Euastacus australasiensis*	NSW	Cuthill et al.^4^
*Temnohaswellia umbella*	KX095318	*Euastacus guwinus*	NSW	Cuthill et al.^4^
*Temnohaswellia verruca*	KX095319–KX095323, KX096325	*Euastacus reductus, E. polysetosus, E. gamilaroi, E. dharawalus, E. yanga, E. brachythorax*	NSW	Cuthill et al.^4^
*Didymorchis* sp.	AY157163	*Cherax quadricarinatus*	QLD	Lockyer et al.^24^
Temnocephalidae sp.	MW136160–MW136166	*Macrobrachium australiense*	NSW	This study

Fifty-three nuclear DNA 28S RNA sequences were obtained from GenBank and subjected to phylogenetic analysis, with *Didymorchis* sp. (AY157163) set as the outgroup. Sequences were aligned using the MUSCLE algorithm in MEGA-X^14^ where they were then exported for manual adjustments using BioEdit^15^. The alignment was then truncated to 614 bp, based on the shortest sequence. Alignment gaps were removed from analysis. The final alignment was uploaded as supplementary file1. The aligned and adjusted sequence files were then converted to NEXUS format using ALTER^16^ to determine the best model for Bayesian analysis using jModelTest 2.0^17^. The NEXUS files were converted again using ALTER^16^ to be compatible with Bayesian analysis software, MrBayes 3.2^18^, using GTR + I + G as the best fit evolutionary model. Analysis was run for 2,000,000 generations with temp = 0.05. The first 25% of the run is discarded as burnin. All other parameters were kept as the default values of the program. Following the conclusion of Bayesian analysis, the resulting tree file was visualised using FigTree 1.4.4^19^ and edited with Adobe Illustrator.

## Results

### Prevalence and distribution

A total of 54 common Australian river prawn, *M. australiense,* were examined for evidence of infection with Temnocephalidae species, of which 96.3% showed at least one sign of infection with Temnocephalidae. Among them, 13 were infected with both eggs and adults whereas 39 were infected with eggs only. A map of the study area and location of sampling site where Temnocephalidae species were located is given in Fig. 1.

### Identification

**Figure 2:**
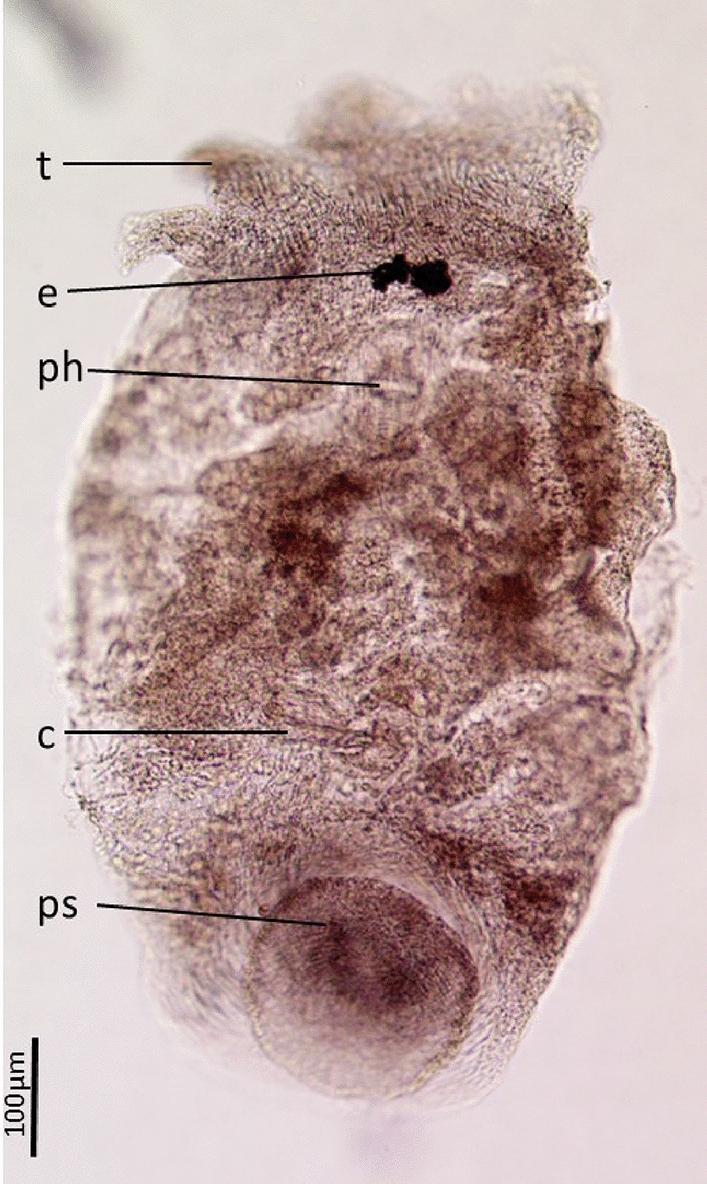
Microscopic image of a Temnocaphalidae found in the present study. Abbreviation includes: *C* cirrus, *E* eyespot, *Ph* pharynx, *Ps* posterior sucker, *T* tentacle.

Thirteen Temnocephalidae worms were collected from *M. australiense*. Due to damage and immaturity of the worms collected from *M. australiense,* no useful diagnostic features existed with which to identify the genus or species of the worms. Therefore, they have been referred to as Temnocephalidae sp. based on the presence of tentacles on the anterior margin of the body, and pedunculate sucker located dorsally on the ventral surface (Fig. 2). In the absence of further specimens, a formal description of this specimen is not provided. Mix of specimens and eggs were deposited at the Australian Museum, under accession numbers: W53508-53516.

### Histopathology

**Figure 3:**
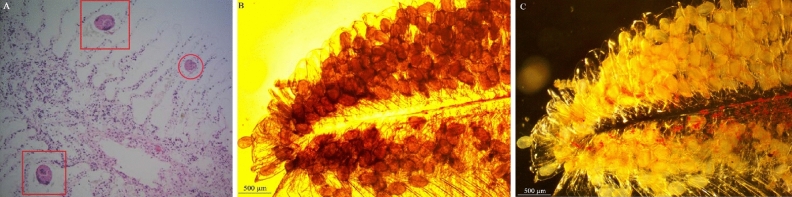
Microscopy of gill arch from *Macrobrachium australiense.* **(A)** Histopathology of the gill arch, noting the Temnocephalan egg (circle), and the appearance of damage to gill tissue surrounding eggs (squares); **(B)** microscopy of gill arch heavily infested with Temnocephalan eggs; **(C)** phase contrast microscopy of gill arch heavily infested with Temnocephalan eggs.

Advanced autolysis interfered with histological interpretation of anatomy infected with Temnocephalidae material. Temnocephalidae eggs appeared as round structures (100–150 µm) between secondary gill lamellae, with thin cuticles and eosinophilic cellular structure lacking orientation (Fig. 3A). Possible mechanical damage to gill lamellae resulting from either egg deposition or autolysis is evident. Heavy infestations of Temnocephalan eggs were present on the gills of *M. australiense* (Fig. 3B,C).

### Phylogeny

**Figure 4:**
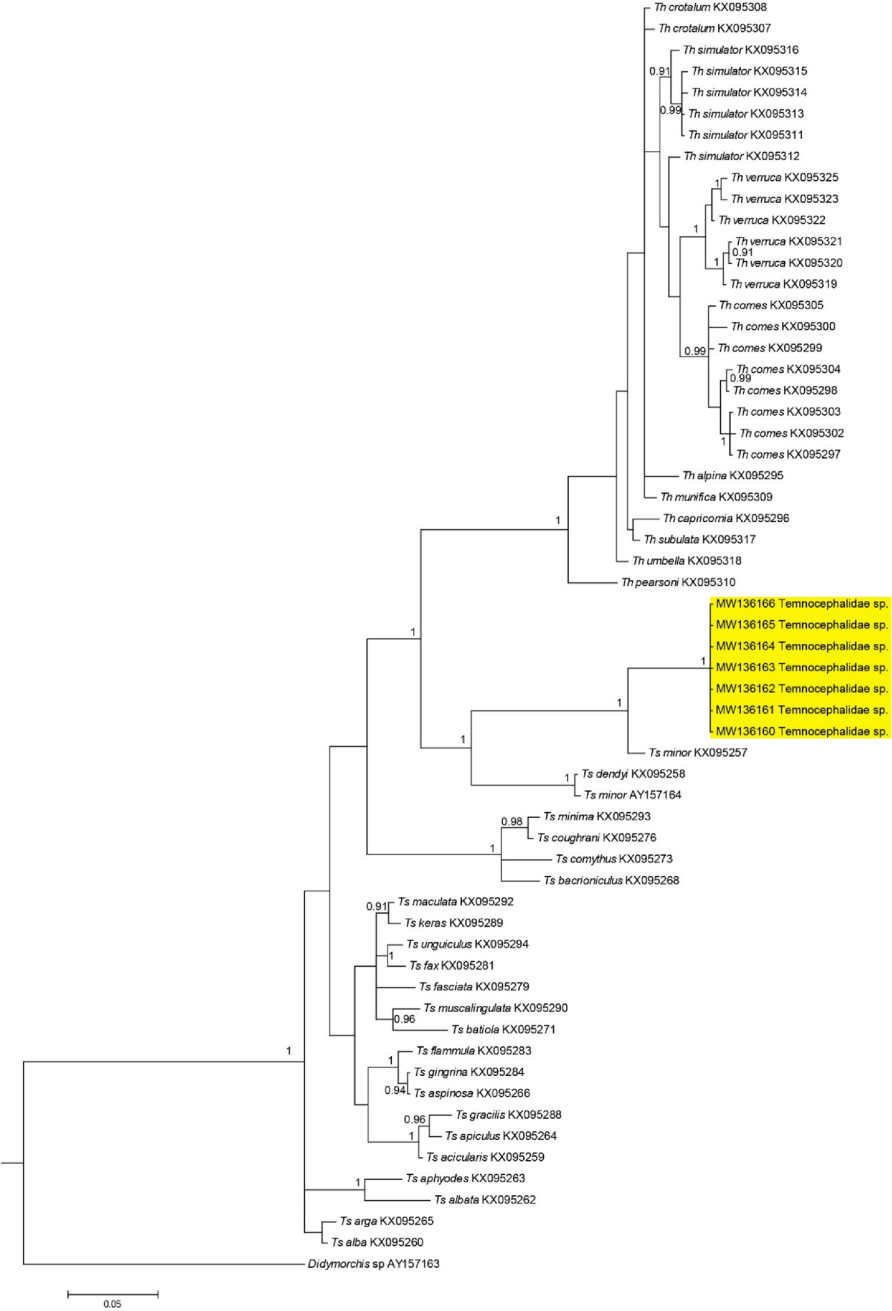
Phylogenetic placement of specimens in the present study based on 28S rRNA sequences, with all available 28S sequences of Temnocephalidae spp. from GenBank. *Didymorchis* sp. was used as an outgroup in this analysis. Bayesian posterior probability values are indicated on the branches.

Seven sequences of the 28S rRNA gene, all 772 base pairs in length, were successfully obtained from Temnocephalidae specimens found as ectosymbionts of *M. australiense.* All Temnocephalidae samples collected in the present study had identical 28S rRNA sequences (Table S1, Fig. 4). Pairwise genetic distances between Temnocephalidae sp. and *Temnosewellia* spp. ranged from 0.04–0.12, and variation between Temnocephalidae sp. and *Temnohaswellia* spp. ranged from 0.14–0.16. In the phylogenetic tree built based on these sequences (Fig. 4), our specimens formed a highly supported clade separated from other Temnocephalidae species reported from Australia.

## Discussion

The present study reveals the first report of Temnocephalidae species in the southern Riverina region of New South Wales, corresponding to the second report of *M. australiense* as a host to taxa within the Temnocephalidae family. There have only been two species of *Temnosewellia* reported as symbionts of *Macrobrachium* species, *Temnosewellia queenslandensis* and *Temnosewellia neqae*, both of which have not been reported below the 28^th^ parallel south^20^. Unfortunately there were no useful diagnostic features available to discern the genus or species due to the immaturity and method of preserving these specimens, as the temperature shock of freezing and thawing likely resulted in the breakdown of the specimens^21^. Due to the opportunistic nature of our sampling, it is difficult to draw definitive conclusions on the relationship between this Temnocephalidae species and its host, although several observations were made. The immaturity of these specimens may indicate that they are unable to survive on *M. australiense* hosts for an extended period; however this conclusion should be approached with caution because this study was based on a single sampling event. The grooming behaviour of *M. australiense* is well established, with experimental studies demonstrating that fouling organisms such as Temnocephalans are quickly removed from the host^22^. The heavy infestations of Temnocephalidae eggs on the gills of *M. australiense*, yet the lack of mature specimens, indicate that this Temnocephalidae species may opportunistically reproduce on *M. australiense* in locations that are not easily groomed by the host, where they are promptly removed by the host shortly after hatching. Observations of the gills showed significant build-up of detritus collecting around the egg capsules, appearing as brown patches within the branchial chamber that were visible to the naked eye. In addition to providing shelter from the grooming behaviour of the host, this possibly provides a favourable environment to the freshly hatched Temnocephalans, as the deposits of detritus within the branchial chamber likely provides a source of nutrition to the Temnocephalans in the form of microflora^5^. Conversely, the build-up of both detritus and Temnocephalan material among the gills and within the branchial chamber may have a negative impact on the respiratory abilities of the host, although heavy infestation with Temnocephalans have never been reported as causes of mortality in crustaceans^23^. Histopathology of gill sections provided little information on the impact of these heavy infestations on *M. australiense* due to autolysis, likely accelerated by the freezing and thawing of the crustaceans prior to examination. There appears to be mechanical damage to the gill lamellae surrounding Temnocephalan eggs, however the degree of damage is uncertain. It is possible that respiration is affected with heavy infestations within the branchial chamber, yet a larger number of fresh samples are required in order to confirm this hypothesis. As this study consisted of a single sample during summer, future surveys of the Mantangery Lagoon sampling site should aim to conduct seasonal surveys to understand how seasonality influences the severity of Temnocephalidae infestation on the host. Examination and dissection of crustaceans should occur while the host is fresh to ensure that damage to both Temnocephalidae and host tissue is minimised, so that a diagnosis of genus and species can be made for the symbionts, and host respiratory structures are maintained for histopathological interpretation. In addition, the relationship between the Temnocephalans and host should be explored in future studies to determine whether mature Temnocephalans are able to colonise the host, or whether the host only acts as a site of reproduction for this species.

Another significant finding includes the genetic characterisation based on 28S rRNA of the Temnocephalan species obtained from *M. australiense*. All samples obtained in the present study had identical sequences in the 28S rRNA region, suggesting that all worms characterised belonged to the same species (Table S1). Consequently, it was found that both KX095258 and AY157164 had identical sequences despite being described as different species. This suggests that one of these sequences has been misidentified, and in the absence of justification for identification of species by Lockyer, et al.^24^, it is likely that AY157164 is actually *Temnosewellia dendyi*, and not *Temnosewellia minor*, based on the 28S rRNA region. The mean genetic distance between groups indicates that our Temnocephalidae sp. is more closely related to *Temnosewellia* spp. (= 0.09) than it is to *Temnohaswellia* spp. (= 0.15). Although, in the absence of morphological data and less than comprehensive comparative molecular sequences, it is unable to be determined systematically where the samples obtained in this study should be placed.

In the phylogenetic tree based on 28S rRNA sequences, there were clear groupings of Temnocephalidae sp. found as symbionts of *M. australiense*, *Temnosewellia* spp., and *Temnohaswellia* spp. The grouping of the samples obtained in this study provides evidence to indicate that they are either a described species with no comparative molecular sequences available, or that they are perhaps a novel species. Furthermore, it should be noted that there is a trend in the grouping of sequences in the tree based on the genera of the host from which they were obtained. This is consistent with previous studies regarding the phylogeny of Australian Temnocephalans^4,25^, suggesting that host-switching is an important driver of speciation and genetic diversity within the Temnocephalidae. This pattern is even more evident when all available 28S rRNA sequences are included in the phylogenetic tree, with for example, *Temnosewellia* species from *Cherax* hosts forming distinct groups when compared to *Temnosewellia* species obtained from *Euastacus* hosts (Fig. 4).

This study also provides an insight into the biodiversity and fauna of the Murrumbidgee catchment area which is home to many wetlands and riverine environments in Australia. Previous research^26–32^ have shown increase in number of invasive and/or pathogenic species in the aquatic systems of the Murrumbidgee catchment. Another interesting area for future studies would be to understand the impact of environmental changes in the Murrumbidgee catchment on the abundance and occurrence of Temnocephalidae species and their hosts.

## Supplementary Information

Supplementary Table S1.

Supplementary Information.

## Data Availability

The data that supports the findings of this study are available from the corresponding author upon request.

## References

[1] Torkkola JJ, Hemsley DW (2019). Prawn parade: n#otes on *Macrobrachium australiense* Holthius, 1950 climbing vertical concrete overflow steps at Gold Creek Reservoir, Queensland. Mar. Freshw. Res..

[2] Rahi ML, Mather PB, Ezaz T, Hurwood DA (2019). The molecular basis of freshwater adaptation in prawns: Insights from comparative transcriptomics of three *Macrobrachium* species. Genome Biol. Evol..

[3] Martínez-Aquino A, Vigliano-relva J, Brusa F, Damborenea C (2017). Historical biogeography of Temnocephalida (Platyhelminthes, Rhabdocoela): Testing the Gondwanan hypothesis. Syst. Biodivers..

[4] Cuthill JFH (2016). Australian spiny mountain crayfish and their temnocephalan ectosymbionts: An ancient association on the edge of coextinction?. Proc. R. Soc. B Biol. Sci..

[5] Cannon LRG, Jennings JB (1987). Occurrence and nutritional relationships of four ectosymbiotes of the freshwater crayfishes *Cherax dispar* Riek and *Cherax punctatus* Clark (Crustacea: Decapoda) in Queensland. Mar. Freshw. Res..

[6] Cannon LRG, Sewell KB (1995). Craspedellinae Baer, 1931 (Platyhelminthes: Temnocephalida) ectosymbionts from the branchial chamber of Australian crayfish (Crustacea: Parastacidae). Mem. Queensl. Mus..

[7] Johnsen BO, Jensen AJ (1986). Infestations of Atlantic salmon, Salmo salar, by Gyrodactylus salaris in Norwegian rivers. J. Fish Biol..

[8] Jalali B, Shamsi S, Barzegar M (2005). Occurrence of Gyrodactylus spp (Monogenea: Gyrodactylidae) from Iranian freshwater fishes. Iran. J. Fisheries Sci..

[9] Sewell K, Cannon LRG, Blair D (2006). A review of *Temnohaswellia* and *Temnosewellia* (Platyhelminthes: Temnocephalida: Temnocephalidae), ectosymbionts from Australian crayfish *Euastacus* (Parastacidae). Mem. Queensl. Mus..

[10] Sewell KB, Cannon LRG (1998). New Temnocephalans from the banchial chamber of Australian *Euastacus* and *Cherax* crayfish hosts. Proc. Linnean Soc. NSW.

[11] Shamsi S, Briand MJ, Justine J-L (2017). Occurrence of *Anisakis* (Nematoda: Anisakidae) larvae in unusual hosts in Southern hemisphere. Parasitol. Int..

[12] Olson P, Cribb T, Tkach V, Bray R, Littlewood D (2003). Phylogeny and classification of the Digenea (Platyhelminthes: Trematoda). Int. J. Parasitol..

[13] Shamsi S (2021). Characterization of *Clinostomum* sp. (Trematoda: Clinostomidae) infecting cormorants in south-eastern Australia. Parasitol. Res..

[14] Kumar S, Stecher G, Li M, Knyaz C, Tamura K (2018). MEGA X: Molecular evolutionary genetics analysis across computing platforms. Mol. Biol. Evol..

[15] Hall TA (1999). BioEdit: A user-friendly biological sequence alignment editor and analysis program for windows 95/98/NT. Nucleic Acids Symp. Ser..

[16] Glez-Pena D, Gomez-Blanco D, Reboiro-Jato M, Fdez-Riverola F, Posada D (2010). ALTER: program-oriented conversion of DNA and protein alignments. Nucleic Acids Res..

[17] Darriba D, Taboada GL, Doallo R, Posada D (2012). jModelTest 2: More models, new heuristics and parallel computing. Nat. Methods.

[18] Ronquist F, Huelsenbeck JP (2003). MrBayes 3: Bayesian phylogenetic inference under mixed models. Bioinformatics.

[19] Rambaut, A. *FigTree v1.4.2, a Graphical Viewer of Phylogenetic Trees*. http://tree.bio.ed.ac.uk/software/figtree/ (2014).

[20] Cannon LRG (1993). New temnocephalans (Platyhelminthes): Ectosymbionts of freshwater crabs of shrimps. Mem. Queensl. Mus..

[21] Sewell, K. B. Key to the genera and checklist of species of Australian temnocephalans (Temnocephalida). *Mus. Victoria Sci. Rep.* **17**, 1–13. 10.24199/j.mvsr.2013.17 (2013).

[22] Jones TC, Lester RJC (1996). Factors influencing populations of the ectosymbiont *Diceratocephala boschmai* (Platyhelminthes; Temnocephalida), on the redclaw crayfish *Cherax quadricarinatus* maintained under laboratory conditions. Aquaculture.

[23] Edgerton BF, Evans LH, Stephens FJ, Overstreet RM (2002). Synopsis of freshwater crayfish diseases and commensal organisms. Aquaculture.

[24] Lockyer AE, Olson PD, Littlewood DTJ (2003). Utility of complete large and small subunit rRNA genes in resolving the phylogeny of the Neodermata (Platyhelminthes): Implications and a review of the cercomer theory. Biol. J. Lin. Soc..

[25] Blair, D. *et al.**History of an association: Temnocephalan flatworms and freshwater crayfish*. (2011).

[26] Zhu X, Barton DP, Wassens S, Shamsi S (2021). Morphological and genetic characterisation of the introduced copepod *Lernaea cyprinacea* Linnaeus (Cyclopoida: Lernaeidae) occurring in the Murrumbidgee catchment, Australia. Mar. Freshw. Res..

[27] Shamsi S, Turner A, Wassens S (2017). Description and genetic characterization of a new *Contracaecum* larval type (Nematoda: Anisakidae) from Australia. J. Helminthol..

[28] Shamsi S, Stoddart A, Smales L, Wassens S (2019). Occurrence of *Contracaecum bancrofti* larvae in fish in the Murray-Darling Basin. J. Helminthol..

[29] Shamsi, S. *et al. Dermocystidium* sp. infection in farmed Murray cod, *Maccullochella peelii*. *Aquaculture* **528**, 735596. 10.1016/j.aquaculture.2020.735596 (2020).

[30] Shamsi S (2021). Wild fish as reservoirs of parasites on Australian Murray Cod farms. Aquaculture.

[31] Rochat EC, Blasco-Costa I, Scholz T, Unmack PJ (2020). High diversity of metazoan parasites in carp gudgeons (Eleotridae: Hypseleotris spp.) from Eastern Australia. J. Helminthol..

[32] Kaminskas S (2020). Alien pathogens and parasites impacting native freshwater fish of southern Australia: A scientific and historical review. Aust. Zool..

