# Supplementary material for: Characterisation of Temnocephalidae flatworms in common Australian freshwater prawn, Macrobrachium australiense
Source: Sci Rep. 2022 Jan 26;12:1396. doi: 10.1038/s41598-022-05123-z (PMC8792052; doi:10.1038/s41598-022-05123-z)
Supplement: Supplementary file 2 — Supplementary Information. [file 41598_2022_5123_MOESM2_ESM.docx]

>Temnocephalidae sp. MW136160

CTGTTGTTACTCGAGAGTCGAATTGTTCGGTATTGCAATTCAAAGTGGGTGGTAGGCTCC

ATCCAAGGCTAAATATAACACTAGACCGATAGCTAACAAGTACCGTGAGGGAAAGTTGAA

AAGAACTTTGAAGAGAGAGTTAATAGTACGTGAAATCACCCAGAGGTAAACGGGTAAAGC

CGAAATTGGTTGAGAGGCTGATGTCTTGTGACC--------TAGTTTGTGGGTTAGAGG-

CGATTTGTTAT---YGAACGTCTCTGATTTAT--------GAGTCTGGGTCTATTGGCTG

TTTCCTCTTTGCCTAA-ACCATGATCGACGATGCGGTYGTTCGTAGTGGGATAAGGTAGC

GATTGGGTCAATT-GRTCCGATTGTGTTATAGATCYCAGAACT-------------G---

-AACAGACTGTGTTGTTGTGCAGTAGCGATGCAAGCC--TTA---------AGGCGCACA

AATTCCGCTGCGGTTGGTGGATGCTTGGCTCACTTGTTTTAGAGTTTGMCGAGTAT-TTC

GCTGRCTGATGGTGGGGTCA---AATTGTGTATTGCATCCATAATCTATGGTTCAATGTA

GGCCATTTACCTGT

>Temnocephalidae sp. MW136161

CTGTTGTTACTCGAGAGTCGAATTGTTCGGTATTGCAATTCAAAGTGGGTGGTAGGCTCC

ATCCAAGGCTAAATATAACACTAGACCGATAGCTAACAAGTACCGTGAGGGAAAGTTGAA

AAGAACTTTGAAGAGAGAGTTAATAGTACGTGAAATCACCCAGAGGTAAACGGGTAAAGC

CGAAATTGGTTGAGAGGCTGATGTCTTGTGACC--------TAGTTTGTGGGTTAGAGG-

CGATTTGTTAT---CGAACGTCTCTGATTTAT--------GAGTCTGGGTCTATTGGCTG

TTTCCTCTTTGCCTAA-ACCATGATCGACGATGCGGTCGTTCGTAGTGGGATAAGGTAGC

GATTGGGTCAATT-GRTCCGATTGTGTTATAGATCCCAGAACT-------------G---

-AACAGACTGTGTTGTTGTGCAGTAGCGATGCAAGCC--TTA---------AGGCGCACA

AATTCCGCTGCGGTTGGTGGATGCTTGGCTCACTTGTTTTAGAGTTTGMCGAGTAT-TTC

GCTGRCTGATGGTGGGGTCA---AATTGTGTATTGCATCCATAATCTATGGTTCAATGTA

GGCCATTTACCTGT

>Temnocephalidae sp. MW136162

CTGTTGTTACTCGAGAGTCGAATTGTTCGGTATTGCAATTCAAAGTGGGTGGTAGGCTCC

ATCCAAGGCTAAATATAACACTAGACCGATAGCTAACAAGTACCGTGAGGGAAAGTTGAA

AAGAACTTTGAAGAGAGAGTTAATAGTACGTGAAATCACCCAGAGGTAAACGGGTAAAGC

CGAAATTGGTTGAGAGGCTGATGTCTTGTGACC--------TAGTTTGTGGGTTAGAGG-

CGATTTGTTAT---CGAACGTCTCTGATTTAT--------GAGTCTGGGTCTATTGGCTG

TTTCCTCTTTGCCTAA-ACCATGATCGACGATGCGGTYGTTCGTAGTGGGATAAGGTAGC

GATTGGGTCAATT-GRTCCGATTGTGTTATAGATCYCAGAACT-------------G---

-AACAGACTGTGTTGTTGTGCAGTAGCGATGCAAGCC--TTA---------AGGCGCACA

AATTCCGCTGCGGTTGGTGGATGCTTGGCTCACTTGTTTTAGAGTTTGMCGAGTAT-TTC

GCTGRCTGATGGTGGGGTCA---AATTGTGTATTGCATCCATAATCTATGGTTCAATGTA

GGCCATTTACCTGT

>Temnocephalidae sp. MW136163

CTGTTGTTACTCGAGAGTCGAATTGTTCGGTATTGCAATTCAAAGTGGGTGGTAGGCTCC

ATCCAAGGCTAAATATAACACTAGACCGATAGCTAACAAGTACCGTGAGGGAAAGTTGAA

AAGAACTTTGAAGAGAGAGTTAATAGTACGTGAAATCACCCAGAGGTAAACGGGTAAAGC

CGAAATTGGTTGAGAGGCTGATGTCTTGTGACC--------TAGTTTGTGGGTTAGAGG-

CGATTTGTTAT---YGAACGTCTCTGATTTAT--------GAGTCTGGGTCTATTGGCTG

TTTCCTCTTTGCCTAA-ACCATGATCGACGATGCGGTYGTTCGTAGTGGGATAAGGTAGC

GATTGGGTCAATT-GRTCCGATTGTGTTATAGATCYCAGAACT-------------G---

-AACAGACTGTGTTGTTGTGCAGTAGCGATGCAAGCC--TTA---------AGGCGCACA

AATTCCGCTGCGGTTGGTGGATKCTTGGCTCACTTGTTTTAGAGTTTGACGAGTAT-TTC

GCTGACTGATGGTGGGGTCA---AATTGTGTATTGCATCCATAATCTATGGTTCAATGTA

GGCCATTTACCTGT

>Temnocephalidae sp. MW136164

CTGTTGTTACTCGAGAGTCGAATTGTTCGGTATTGCAATTCAAAGTGGGTGGTAGGCTCC

ATCCAAGGCTAAATATAACACTAGACCGATAGCTAACAAGTACCGTGAGGGAAAGTTGAA

AAGAACTTTGAAGAGAGAGTTAATAGTACGTGAAATCACCCAGAGGTAAACGGGTAAAGC

CGAAATTGGTTGAGAGGCTGATGTCTTGTGACC--------TAGTTTGTGGGTTAGAGG-

CGATTTGTTAT---YGAACGTCTYTGATTTAT--------GAGTCTGGGTCTATTGGCTG

TTTCCTCTTTGCCTAA-ACCATGATCGACGATGCGGTCGTTCGTAGTGGGATAAGGTAGC

GATTGGGTCAATT-GRTCCGATTGTGTTATAGATCCCAGAACT-------------G---

-AACAGACTGTGTTGTTGTGCAGTAGCGATGCAAGCC--TTA---------AGGCGCACA

AATTCCGCTGCGGTTGGTGGATKCTTGGCTCACTTGTTTTAGAGTTTGMCGAGTAT-TTC

GCTGRCTGATGGTGGGGTCA---AATTGTGTATTGCATCCATAATCTATGGTTCAATGTA

GGCCATTTACCTGT

>Temnocephalidae sp. MW136165

CTGTTGTTACTCGAGAGTCGAATTGTTCGGTATTGCAATTCAAAGTGGGTGGTAGGCTCC

ATCCAAGGCTAAATATAACACTAGACCGATAGCTAACAAGTACCGTGAGGGAAAGTTGAA

AAGAACTTTGAAGAGAGAGTTAATAGTACGTGAAATCACCCAGAGGTAAACGGGTAAAGC

CGAAATTGGTTGAGAGGCTGATGTCTTGTGACC--------TAGTTTGTGGGTTAGAGG-

CGATTTGTTAT---YGAACGTCTCTGATTTAT--------GAGTCTGGGTCTATTGGCTG

TTTCCTCTTTGCCTAA-ACCATGATCGACGATGCGGTYGTTCGTAGTGGGATAAGGTAGC

GATTGGGTCAATT-GRTCCGATTGTGTTATAGATCYCAGAACT-------------G---

-AACAGACTGTGTTGTTGTGCAGTAGCGATGCAAGCC--TTA---------AGGCGCACA

AATTCCGCTGCGGTTGGTGGATGCTTGGCTCACTTGTTTTAGAGTTTGMCGAGTAT-TTC

GCTGRCTGATGGTGGGGTCA---AATTGTGTATTGCATCCATAATCTATGGTTCAATGTA

GGCCATTTACCTGT

>Temnocephalidae sp. MW136166

CTGTTGTTACTCGAGAGTCGAATTGTTCGGTATTGCAATTCAAAGTGGGTGGTAGGCTCC

ATCCAAGGCTAAATATAACACTAGACCGATAGCTAACAAGTACCGTGAGGGAAAGTTGAA

AAGAACTTTGAAGAGAGAGTTAATAGTACGTGAAATCACCCAGAGGTAAACGGGTAAAGC

CGAAATTGGTTGAGAGGCTGATGTCTTGTGACC--------TAGTTTGTGGGTTAGAGG-

CGATTTGTTAT---YGAACGTCTCTGATTTAT--------GAGTCTGGGTCTATTGGCTG

TTTCCTCTTTGCCTAA-ACCATGATCGACGATGCGGTYGTTCGTAGTGGGATAAGGTAGC

GATTGGGTCAATT-GRTCCGATTGTGTTATAGATCYCAGAACT-------------G---

-AACAGACTGTGTTGTTGTGCAGTAGCGATGCAAGCC--TTA---------AGGCGCACA

AATTCCGCTGCGGTTGGTGGATKCTTGGCTCACTTGTTTTAGAGTTTGMCGAGTAT-TTC

GCTGACTGATGGTGGGGTCA---AATTGTGTATTGCATCCATAATCTATGGTTCAATGTA

GGCCATTTACCTGT

>Ts_minor_KX095257

CTGATGTTACTCGAGAGTCGAATTGTTCGGTATTGCAATTCAAAGTGGGTGGTAGGCTCC

ATCCAAGGCTAAATATAACACTAGACCGATAGCTAACAAGTACCGTGAGGGAAAGTTGAA

AAGAACTTTGAAGAGAGAGTTAATAGTACGTGAAATCACCCAGAGGTAAACGGGTAAAGC

CGAAATTGGTTGAGAGGCTGATGTCTTGTAGCC--------TGGTTTGTGGTTCGGAGG-

CGATTTGTTAT---CGAACGTCTTTGGGCCAT--------GAGTTTGGGTTATTTGGCTG

TTTCCTCTTTGCCTAA-ACCATGATCGACGATGCGGTCGTTCGTAGTGGGAGAAGGTAGC

GATTGAGTCAATT-GATTCGATTGTATTATAGCTCCCAGAACT-------------G---

-AACAGACTGTGTTGTTGTGCAGTAGCGATGCAAGCC--TTA---------AGGCGCACA

AATTTTGCTGCGGTTGGTGGATGCTTGGCTCACTTGTTTTAGAGTTTGTCGAGTAT-TTC

GCTGACTGATGGTGGAGTCA----ATTGTGTATTGCATCCATAATCTATGGTTCAATGTA

GGCCATTTACCTGT

>Ts_minor_AY157164

CTAAAGGTACTCCAGAGTCGAATTGTTCGGTATTGCAATTCAAAGTGGGTGGTAGGCTCC

ATCCAAGGCTAAATATAACACTAGACCGATAGTTAACAAGTACCGTGAGGGAAAGTTGAA

AAGAACTTTGAAGAGAGAGTTAATAGTACGTGAAATCACCCAGAGGTAAACGGGTAAAGC

CGAAATTGGTAGAGAGGCTGATGTCTTCCGGTT-------TGTGCATGCGGTTTGGAGG-

CGATTTGTTTT---AAAACGTCTTTGGGCTGT--------GTGTTATGGCTGGTTGGCTG

TCTCCTTTTTGCTTGA-ACCATGATCGACGATGTGGTCGTTCGTAGTGGGAGAAGGTAGC

GTTTGAGCCATTT-GGTTCGATTGTATTATAGCTCCCATATTT-------------G---

-AACAGACTGTGTTGTTGTGCAGTAGCGATGTAAGCC--TTA---------AGGCGCACA

AATTCTGCTGCAGTTGGCGGGTGCTTGGCTCACTTGTTTTAGAGTTTGCTGAGTGC-AGT

GCTGGCTGATGGTGGGGTAA---AATTGTGCATTACATCTATAATCTATGGTTTAATGTA

GGCCATTTACCTGT

>Ts_dendyi_KX095258

CTAAAGGTACTCCAGAGTCGAATTGTTCGGTATTGCAATTCAAAGTGGGTGGTAGGCTCC

ATCCAAGGCTAAATATAACACTAGACCGATAGTTAACAAGTACCGTGAGGGAAAGTTGAA

AAGAACTTTGAAGAGAGAGTTAATAGTACGTGAAATCACCCAGAGGTAAACGGGTAAAGC

CGAAATTGGTAGAGAGGCTGATGTCTTCCGGTT-------TGTGCATGCGGTTTGGAGG-

CGATTTGTTTT---AGAACGTCTTTGGGCTGT--------GTGTTATGGCTGGTTGGCTG

TCTCCTTTTTGCTTGA-ACCATGATCGACGATGTGGTCGTTCGTAGTGGGAGAAGGTAGC

GTTTGAGCCATTT-GGTTCGATTGTATTATAGCTCCCATATTT-------------G---

-AACAGACTGTGTTGTTGTGCAGTAGCGATGTAAGCC--TTA---------AGGCGCACA

AATTCTGCTGCAGTTGGCGGGTGCTTGGCTCACTTGTTTTAGAGTTTGCTGAGTGC-AGT

GCTGGCTGATGGTGGGGTAA---AATTGTGCATTACATCTATAATCTATGGTTTAATGTA

GGCCATTTACCTGT

>Ts_acicularis_KX095259

CTGAAGTTACTCCAGAGTCGAATTGTTCGGTATTGCAATTCAAAGCGGGTGGTAGGCTCC

ATCCAAGGCTAAATATAACACTAGACCGATAGTTAACAAGTACCGTGAGGGAAAGTTGAA

AAGAACTTTGAAGAGAGAGTTAATAGTACGTGAAATCACCCAGAGGTAAACGGGTAAAGC

TGAAATTGGCTGAGAGGCTGATGTCTTCTGGTA------CTCGGTATGCAGTGTGGAGGT

CGATTTGTTTT---AGAACGTCTCTGTGCTGTTTGTTA--GTATTCGAGCTAGTTGACTG

TTTCCTCTTAGCCTTA-ACCATGATCGACGATGCGGTCGTTTGTGGTGAGAGAAGGTAGC

GATTGGGTCCTTGCGGCTTGATTGTATTATAGCTCTCACCAGCAT---------TTG---

-AACAGACTGTGTTGTTGTGCAGTAGCGATGTGAGCC--TTA---------AGGCGCACA

AATTCTGCTGCGGTTGGTGGGCGCTTGACTTACTTGTTTTAGAGTTTGTTGAGTGC-AGC

GCTGGCTGTTGGTGGAGTCAACTAATTGTGCATCACATCCATAATCTATGGTTTAATGTA

GGCCATTTACCCGT

>Ts_alba_KX095260

CTGAAGTTACTCCAGAGTCGAATTGTTCGGTATTGCAATTCAAAGTGGGTGGTAGGCTCC

ATCCAAGGCTAAATATAACACTAGACCGATAGTTAACAAGTACCGTGAGGGAAAGTTGAA

AAGAACTTTGAAGAGAGAGTTAATAGTACGTGAAATCACCCAGAGGTAAACGGGTAAAGC

CGAAATTGGCAGGGAGGCTGATGTCTTCTGGTA--------CGGTATGCAGCGTGGAGGT

CGATTTGTTTC---TGAACGTCTCTGTGCTGTTT--TA--GTATTCGTGCTAGTTGGCTG

TTTCCTCTTTGCCTTA-ACCATGATCGACGATGCGGTCGTTTGTAGTGGGAGAAGGTAGC

GATTGGACCATTTCGGTTTGATCGTGTTATAGCTCTCA--TGAAT--------TTTG---

-AACAGACTGTGTTGTTGTGCAGTAGCGATGTGAGCC--TTA---------AGGCGCACA

AATTCTGCTGCGGTTGGTGGGCGCTTGACTTACTTGTTTTAGAGTTTGTTGAGTGC-AGC

GCTGGCTGTTGGCGGGGTCT----ATTGTGTATCACATCCATAATCTATGGTTTAATGTA

GGCCATTTACCTGT

>Ts_albata_KX095262

CTGAAGTTACTCGAGAGTCGAATTGTTCGGTATTGCAATTCAAAGCGGGTGGTAGGCTCC

ATCCAAGGCTAAATATAACACCAGACCGATAGTTAACAAGTACCGTGAGGGAAAGTTGAA

AAGAACTTTGAAGAGAGAGTTAATAGTACGTGAAATCACCCAGAGGTAAACGGGTAAAGC

CGAAATTGGCAAGGAGGCTGATGTTTTCTGGTC-------TCGGTATGCAGTGTGGAGGT

CGATTTGTTAT---AAAACGTCTCTATGCTGTTT--TA--GTATCTGAGCTATTTGGCTG

TTTCCTCTTTGTCTTA-ACCATGATCGACGATATAATCGTTTGTAGTGGGAGAAGGTAGC

GATTGAACAATTTCTGTTTGATTGTGTTATAGCTCTCA--TGAAT--------TTTG---

-AACAGATTGTATTGTTGTGCATTAGCGATGTGAGCC--TTT---------AGGCGCACA

AATTTTGCTGCGGTTGGTGGGCACTTGGCTTACTTGTTTTAGAGTTTGCTGAGTGC-AGC

GCTGGCTGTTGGTGAAGTCA-----TTGTGTATCACATCCATAATCTATGGTTTAATGTA

GGCTATTTACCTGT

>Ts_arga_KX095265

CTGAAGTTACTCCAGAGTCGAATTGTTCGGTATTGCAATTCAAAGTGGGTGGTAGGCTCC

ATCCAAGGCTAAATATAACACTAGACCGATAGTTAACAAGTACCGTGAGGGAAAGTTGAA

AAGAACTTTGAAGAGAGAGTTAATAGTACGTGAAATCACCCAGAGGTAAACGGGTAAAGC

CGAAATTGGCAGGGAGGCTGATGCCTTCTGGTA--------CGGTATGCAGCGTGGAGGT

CGATTTGTTTT---TGAACGTCTCTTTGCTGTTT--TA--GTATTCGTGCTAGTTGGCTG

TTTCCTCTTTGCCTTA-ACCATGATCGACGATGCGGTCGTTTGTAGTGGGAGAAGGTAGC

GATTGGACCAATTCGGTTTGATCGTGTTATAGCTCTCA--TGATT--------TTTG---

-AACAGACTGTGTTGTTGTGCAGTAGCGATGTGAGCC--TTA---------AGGCGCACA

AATTCTGCTGCGGTTGGTGGGCGCTTGACTTACTTGTTTTAGAGTTTGTTGAGTGC-AGC

GCTGGCTGTTGGCGGGGTCA---TATTGTGTATCACATCCATAATCTATGGTTTAATGTA

GGCCATTTACCTGT

>Ts_aphyodes_KX095263

CTGAAATTACTCGAGAGTCGAATTGTTCGGTATTGCAATTCAAAGTGGGTGGTAGGCTCC

ATCCAAGGCTAAATATAACACCAGACCGATAGTTAACAAGTACCGTGAGGGAAAGTTGAA

AAGAACTTTGAAGAGAGAGTTAATAGTACGTGAAATCACCCAGAGGTAAACGGGTAAAGC

CGAAATTGGCAGGGAGGCTGATGTCTTCTGGTC-------TCGGTATGCAGTGTAGAGGT

CGATTTGTTTT---AGAACGTCTCTGTGCTGTTT--TA--GTATTTGAGCTAGTTGGCTG

TTTCCTCTTTGTCTTA-ACCATGATCGACGATACGATCGTTTGTAGTGGGAGAAGGTAGC

GATTGGATCATTTCGGTTCGATCGTGTTATAGCTCCCA--TGAAT--------TTTG---

-AACAGATTGTGTTGTTGTGCAGTAGCGATGTGAGCC--GTT---------TGGCGCACA

AATTTTGCTGCGGTTGGTGGGCACTTGGCTTACTTGTTTTAGAGTTTGCTGAGTGC-AAC

GCTGGCTGTTGGTGAAGTCA-----TTGTGTATCACGTCCATAATCTATGGTTTGATGTA

GGCCATTTACCTGT

>Ts_flammula_KX095283

CTGAAATTACTCCAGAGTCGAATTGTTCGGTATTGCAATTCAAAGCGGGTGGTAGGCTCC

ATCCAAGGCTAAATATAACACTAGACCGATAGTTAACAAGTACCGTGAGGGAAAGTTGAA

AAGAACTTTGAAGAGAGAGTTAATAGTACGTGAAATCACCCAGAGGTAAACGGGTAAAGC

CGAAATTGGCTGAGAGGCTGATGTCTTCTGGTT--------CGGTATGCAGTGCGGAGGT

CGATTTGTTTT---AGAACGTCTCTGTGTTGTTTTTAAA-GTATTCGAGCTAGTTGGCTG

TTTCCTCTTTGCCTTA-ACCATGATCGACGATGCGGTCGTTTGTAGTGGGAGAAGGTAGC

GATTGGGTCATTTTGGCCTGATTGTGTTATAGCTCTCATTAACAT--------TTTG---

-AACAGACTGTGTTGTTGTGCAGTAGCGATGTGAGCC--TCA---------AGGCGCACA

AACTTTGCTGCGGTTGGTGGGCGCTTGACTTACTTGTTTTAGAGTTTGTTGAGTGC-AGC

GCTGGCTGTTGGCGGAGTCA-TTCATTGTGCATCACATCCATAATCTATGGTTTAATGTA

GGCCATTTACCCGT

>Ts_apiculus_KX095264

CTGAAGTTACTCCAGAGTCGAATTGTTCGGTATTGCAATTCAAAGCGGGTGGTAGGCTCC

ATCCAAGGCTAAATATAACACTAGACCGATAGTTAACAAGTACCGTGAGGGAAAGTTGAA

AAGAACTTTGAAGAGAGAGTTAATAGTACGTGAAATCACCCAGAGGTAAACGGGTAAAGC

TGAAATTGGCTGAGAGGCTGATGTCTTCTGGTT------TTCGGTATGCAGTGTGGAGGT

CGATTTGTTTT---AGAACGTCTCTGTGCTGTTT--TA--GTATTCGAGCTAGTTGACTG

TTTCCTCTTAGCCTTA-ACCATGATCGACGATGCGGTCGTTTGTGGTGAGAGAAGGTAGC

GATTGAGTCCTTGCGGCTTGATTGTATTATAGCTCTCACCAGCACATTC---ATTTG---

-AACAGACTGTGTTGTTGTGCAGTGGCGATGTGAGCC--TTA---------AGGCGCACA

AATTCTGCTGCGGTTGGTGGGCGCTTGACTTACTTGTTTTAGAGTTTGTTGAGTGT-AGC

GCTGGCTGTTGGTGGAGTCAACTAATTGTGCATCACATCCATAATCTATGGTTTAATGTA

GGCCATTTACCCGT

>Ts_gingrina_KX095284

CTGAAGTTACTCCAGAGTCGAATTGTTCGGTATTGCAATTCAAAGCGGGTGGTAGGCTCC

ATCCAAGGCTAAATATAACACTAGACCGATAGTTAACAAGTACCGTGAGGGAAAGTTGAA

AAGAACTTTGAAGAGAGAGTTAATAGTACGTGAAATCACCCAGAGGTAAACGGGTAAAGC

CGAAATTGGCTGAGAGGCTGATGTCTTCTGGTT--------CGGTATGCAGTGCGGAGGT

CGATTTGTTTT---AGAACGTCTCTGTGCTGTTTTCAAATGTATTCGAGCTAGTTGGCTG

TTTCCTCTTAGCCTTA-ACCATGATCGACGATGCGGTCGTTTGTAGTGGGAGAAGGTAGC

GATTGGGTCATTTTGGCTTGATTGTGTTATAGCTCTCATTAAT-T--------TTTG---

-AACAGACTGTGTTGTTGTGCAGTAGCGATGTGAGCC--TCA---------AGGCGCACA

AACTTTGCTGCGGTTGGTGGGCGCTTGACTTACTTGTTTTAGAGTTTGTTGAGTGC-AGC

GCTGGCTGTTGGCGGAGTCA-TTCATTGTGCATCACATCCATAATCTATGGTTTAATGTA

GGCCATTTACCCGT

>Ts_aspinosa_KX095266

CTGAAGTTACTCCAGAGTCGAATTGTTCGGTATTGCAATTCAAAGCGGGTGGTAGGCTCC

ATCCAAGGCTAAATATAACACTAGACCGATAGTTAACAAGTACCGTGAGGGAAAGTTGAA

AAGAACTTTGAAGAGAGAGTTAATAGTACGTGAAATCACCCAGAGGTAAACGGGTAAAGC

CGAAATTGGCTGAGAGGCTGATGTCTTCTGGTT--------CGGTATGCAGTGCGGAGGT

CGATTTGTTTT---AGAACGTCTCTGTGCTGTTTTCAAATGTATTCGAGCTAGTTGGCTG

TTTCCTCTTAGCCTTA-ACCATGATCGACGATGCGGTCGTTTGTAGTGGGAGAAGGTAGC

GATTGGGTCATTTTGGCTTGATTGTGTTATAGCTCTCATTAAT-T--------TTTG---

-AACAGACTGTGTTGTTGTGCAGTAGCGATGTGAGCC--TCA---------AGGCGCACA

AACTTTGCTGCGGTTGGTGGGCGCTTGACTTACTTGTTTTAGAGTTTGTTGAGTGC-AGC

GCTGGCTGTTGGCGGAGTCA-TTCATTGTGCATCACATCCATAATCTATGGTTTAATGTA

GGCCATTTACCCGT

>Ts_fasciata_KX095279

CTGAAGTTACTCCAGAGTCGAATTGTTCGGTATTGCAATTCAAAGCGGGTGGTAGGCTCC

ATCCAAGGCTAAATATAACACTAGACCGATAGTTAACAAGTACCGTGAGGGAAAGTTGAA

AAGAACTTTGAAGAGAGAGTTAATAGTACGTGAAATCACCCAGAGGTAAACGGGTAAAGC

CGAAATTGGCTGAGAGGCTGATGTCTTCTGGTA-------TCGGCATGCAGTGCGGAGGT

CGATTTGTTTG---AGAACGTCTCTGTGCTGTTC--AAT-GTGTTCGAGCTAGTTGGCTG

TTTCCTCTTTGTCTTA-ACCATGATCGACGATGCGGTCGTTTGTAGTGAGAGAAGGTAGC

GATTGGGTCATTTCGGCTCGATTGTATTATAGCTCTCA-TTAACT--------TTTG---

-AACAGACTGTGTTGTTGTGCAGTAGCGATGTGAGCC--TCT---------AGGCGCACA

AATTCTGCTGCGGTTGGTGGGCGCTTGACTTACTTGTTTTAGAGTTTGTTGAGTGC-AGC

GCTGGCTGTTGGTAGAGTCA-TT--TTGTGTATCACATCCATAATCTATGGTTTAATGTA

GGCCATTTACCCGT

>Ts_gracilis_KX095288

CTGAAGTTACTCCAGAGTCGAATTGTTCGGTATTGCAATTCAAAGCGGGTGGTAGGCTCC

ATCCAAGGCTAAATATAACACTAGACCGATAGTTAACAAGTACCGTGAGGGAAAGTTGAA

AAGAACTTTGAAGAGAGAGTTAATAGTACGTGAAATCACCCAGAGGTAAACGGGTAAAGC

TGAAATTGGCTGAGAGGCTGATGTCTTCTGGTT------TTCGGTATGCAGTGTGGAGGT

CGATTTGTTTT---TGAACGTCTCCGTGCTGTTT--TA--GTACTCGAGCTAGTTGACTG

TTTCCTCTTAGCCTTA-ACCATGATCGACGATGCGGTCGTTTGTGGTGAGAGAAGGTAGC

GATTGGGTCCTTGCGGCTTGATTGTATTATAGCTCTCACCAGCAT--------TTGG---

-AACAGACTGTGTTGTTGTGCAGTGGCGATGTGAGCC--TTA---------AGGCGCACA

AATTCTGCTGCGGTTGGTGGGCGCTTGACTTACTTGTTTCAGAGTTTGTTGAGTGC-AGC

GCTGGCTGTTGGTGGAGTCAACTAATTGTGCATCACATCCATAATCTATGGTTTAATGTA

GGCCATTTACCTGT

>Ts_keras_KX095289

CTGAAGTTACTCCAGAGTCGAATTGTTCGGTATTGCAATTCAAAGCGGGTGGTAGGCTCC

ATCCAAGGCTAAATATAACACTAGACCGATAGTTAACAAGTACCGTGAGGGAAAGTTGAA

AAGAACTTTGAAGAGAGAGTTAATAGTACGTGAAATCACCCAGAGGTAAACGGGTAAAGC

CGAAATTGGCTGAGAGGCTGATGTCTTCTGGTA-------TCGGTATGCAGTGCGGAGGT

CGATTTGTTTT---AAAACGTCTCTGTGTTGTTT--TA--GTATTCGAGCTAGTTGGCTG

TTTCCTCTTTGCCTTA-ACCATGATCGACGATGTGGTCGTTTGTAGTGAGAGAAGGTAGC

GATTGGGTCATTTCGGCCTGATTGTGTTATAGCTCTCAACTAATT--------TTTG---

-AACAGACTGTGTTGTTGTGCAGTAGCGATGTGAGCC--TCT---------AGGCGCACA

AATTCTGTTGCGGTTGGTGGGCGCTTGACTTACTTGTTTTAGAGTTTGTTGAGTGC-AGC

GCTGGCTGTTGGCGGAGTCA-TTCATTGTGTATCACATCCATAATCTATGGTTTAATGTA

GGCCATTTACCCGT

>Ts_maculata_KX095292

CTGAAGTTACTCCAGAGTCGAATTGTTCGGTATTGCAATTCAAAGCGGGTGGTAGGCTCC

ATCCAAGGCTAAATATAACACTAGACCGATAGTTAACAAGTACCGTGAGGGAAAGTTGAA

AAGAACTTTGAAGAGAGAGTTAATAGTACGTGAAATCACCCAGAGGTAAACGGGTAAAGC

CGAAATTGGCTGAGAGGCTGATGTCTTCTGGTA-------TCGGTATGCAGTGCGGAGGT

CGATTTGTTTT---AAAACGTCTCTGTGTTGTTT--TA--GTATTCGAGCTAGTTGGCTG

TTTCCTCTTTGCCTTA-ACCATGATCGACGATGCGGTCGTTTGTAGTGAGAGAAGGTAGC

GATTGGGTCATTTCGGCCTGATTGTGTTATAGCTCTCAACTAACT--------TTTG---

-AACAGACTGTGTTGTTGTGCAGTAGCGATGTGAGCC--TCT---------AGGCGCACA

AATTCTGCTGCGGTTGGTGGGCGCTCGACTTACTTGTTTTAGAGTTTGTTGAGTGC-AGC

GCTGGCTGTTGGCGGAGTCA-TTCATTGTGTATCACATCCATAATCTATGGTTTAATGTA

GGCCATTTACCCGT

>Ts_unguiculus_KX095294

CTGAAGTTACTCCAGAGTCGAATTGTTCGGTATTGCAATTCAAAGCGGGTGGTAGGCTCC

ATCCCAGGCTAAATATAACACTAGACCGATAGTTAACAAGTACCGTGAGGGAAAGTTGGA

AAGAACTTTGAAGAGAGAGTTAATAGTACGTGAAATCACCCAGAGGTAAACGGGTAAAGC

CGAAATTGGCTGAGAGGCTGATGTCTTCTGGTA-------TCGGTATGCGGTGCGGAGGT

CGATTTGTTTT---AAAACGTCTCTGTGCTGTTT--TG--GTATTCGAGCTAGTTGGCTG

TTTCCTCTTTGCCTTA-ACCATGATCGACGATGCGGTCGTTTGTAGTGAGAGAAGGTAGC

GATTGGGTCTTTTCGGCTTGATTGTATTATAGCTCTCAACTAACT--------CTTG---

-AACAGACTGTGTTGTTGTGCAGTAGCGATGTGAGCC--TCTT--------TGGCGCACA

AATTCTGCTGCGGTTGGTGGGCGCTTGACTTACTTGTTTTAGAGTTTGTTGAGTGC-AGC

GCTGGCTGTTGGCGGAGTCA-TTCATTGTGTATCACATCCATAATCTATGGTTTAATGTA

GGCCATTTACCCGT

>Ts_fax_KX095281

CTGAAGTTACTCCAGAGTCGAATTGTTCGGTATTGCAATTCAAAGCGGGTGGTAGGCTCC

ATCCAAGGCTAAATATAACACTAGACCGATAGTTAACAAGTACCGTGAGGGAAAGTTGAA

AAGAACTTTGAAGAGAGAGTTAATAGTACGTGAAATCACCCAGAGGTAAACGGGTAAAGC

CGAAATTGGCTGAGAGGCTGATGTCTTCTGGTA-------TCGGTATGCGGTGCGGAGGT

CGATTTGTGTT---AAAACGTCTCTGTGCTGTTT--TA--GTATTCGAGCTAGTTGGCTG

TTTCCTCTTTGCCTTA-ACCATGATCGACGATGCGGTCGTTTGTAGTGAGAGAAGGTAGC

GATTGGGTCTTTACGGCTCGATTGTGTTATAGCTCTCAACTAACT--------CTTG---

-AACAGACTGTGTTGTTGTGCAGTAGCGATGTGAGCCAATT----------TGGCGCACA

AATTCTGCTGCGGTTGGTGGGCGCTTGACTTACTTGTTTTAGAGTTTGTTGAGTGC-AGC

GCTGGCTGTTGGCGGAGTCA-TTCATTGTGTATCACATCCATAATCTATGGTTTAATGTA

GGCCATTTACCCGT

>Ts_bacrioniculus_KX095268

CTGAAGTTACTCCAGAGTCGAATTGTTCGGTATTGCAATTCAAAGCGGGTGGTAGGCTCC

ATCCAAGGCTAAATATAACACTAGACCGATAGTTAACAAGTACCGTGAGGGAAAGTTGAA

AAGAACTTTGAAGAGAGAGTTAATAGTACGTGAAATCACCCAGAGGTAAACGGGTAAAGC

CGAAATTGGCTGAGAGGCTGATGTCTTCTGGTGCAT---CTCGGTATGCGGTGCGGAGGT

CGATTTGTTTT---AGAACGTCTCTGTGCCGTTG--TA--GTACTCGAGCCAGTTGGCTG

TTTCCTCTTAGCCTTA-ACCATGATCGACGATGCGGTCGTTTGTAGTGGAGAAAGGTAGC

GAGTGCGGCAATTCGTTGTGCTCGTGTTATAGTCTCCATGTTCAT--------TGTG---

-AACAGACTGTGTTGTTGTGCAGTAGCGATGTGAGCC--TTT---------TGGCGCACA

AACTTTGCTGCAGTCGGTGGGCGCTTGACTTACTTGTTTTAGAGTTTGTTGGGTGC-AGC

GCTGGCTGTCGGTGAGGTCACATTATTGTGTATCACATCCATAATCTATGGTTTAATGTA

GGCCATTTACCTGT

>Ts_batiola_KX095271

CTGAAGTTACTCCAGAGTCGAATTGTTCGGTATTGCAATTCAAAGCGGGTGGTAGGCTCC

ATCCAAGGCTAAATATAACACTAGACCGATAGTTAACAAGTACCGTGAGGGAAAGTTGAA

AAGAACTTTGAAGAGAGAGTTAATAGTACGTGAAATCACCCAGAGGTAAACGGGTAAAGC

CGAAATTGGCTGAGAGGCTGATGTCTTCTGGTTGTC---TTCGGTATGCGGTGTGGAGGT

CGATTCCTTTTGCTAGAACGTCTCTGTGCTGTTA--TA--GTATCCGAGCTAGTTGGCTG

TTTCCTCTTAGCCTTA-ACCATGATCGACGATGCGGTCGTTTGTAGTGAGAGAAGGTAGC

GATTGGGTCATTTCGGCTCGATTGTATTATAGCTCTCAACTAACT--------TTTG---

-AACAGACTGTGTTGTTGGGCAGTGGCGGTGTGAGCC--CTT--ATTTTT-GGGCGCACA

AATTCTGCTGCGGTTGGTGGGCGCTTGACTTACTTGTTTTAGAGTTTGTTGAGTGC-AGC

GCTGGCTGTTGGCGGGGTCT----CTTGTGTATCACATCCATAATCTATGGTTTAATGTA

GGCCATTTACCCGT

>Ts_comythus_KX095273

CTGAAGTTACTCCAGAGTCGAATTGTTCGGTATTGCAATTCAAAGCGGGTGGTAGGCTCC

ATCCAAGGCTAAATATAACACTAGACCGATAGTTAACAAGTACCGTGAGGGAAAGTTGAA

AAGAACTTTGAAGAGAGAGTTAATAGTACGTGAAATCACCCAGAGGTAAACGGGTAAAGC

CGAAATTGGCTGAGAGGCTGATGTCTTCTGGTGTGT---CTCGGTATGCGGTGCGGAGGT

CGATTTGTTTTCA-AGAACGTCTCTGTGTCGTTG--CA--GTACTCGAGCCAGTTGGCTG

TTTCCTCTTTGCCTTA-ACCATGATCGACGATGCGGTCGTTTGCAGTGGAGAAAGGTAGC

GAGTGTGGCAATTCGTCGCGCTCGTGTTATAGTCTCCATGTTCAATGCTTTTTTGTATTG

TAACAGACTGTGTTGTTGTGCAGTGGCGATGTGAGCC--TTCTATGTTTA-GGGCGCACA

AATTCTGCTGCAGTCGGTGGGCGCTTGGCTTACTTGTTTCAGAGTTTGCTGAGTGC-AGC

GCTGGCTGTTGGTGGGGGCA----AATGTGCATCACATCCATAATCTATGGTTTAATGTA

GGCCATTTACCTGT

>Ts_coughrani_KX095276

CTGAAGTTACTCCAGAGTCGAATTGTTCGGTATTGCAATTCAAAGCGGGTGGTAGGCTCC

ATCCAAGGCTAAATATAACACTAGACCGATAGTTAACAAGTACCGTGAGGGAAAGTTGAA

AAGAACTTTGAAGAGAGAGTTAATAGTACGTGAAATCACCCAGAGGTAAACGGGTAAAGC

CGAAATTGGCTGAGAGGCTGATGTCTTCTGGTGTATTTTCTCGGTATGCGGTGCGGAGGT

CGATTTGTTTT---AGAACGTCTCTGTGCCGTTG--CA--GTACTCGAGCCAGTTGGCTG

TTTCCTCTTAGCCTTA-ACCATGATCGACGATGCGGTCGTTTGTAGTGGAGAAAGGTAGC

GAGTGTGGCATTTTGTCGCGCTTGTGTTATAGTCTCCATGTTCAT--------TGTG---

-AACAGACTGTGTTGTTGTGCAGTGGCGATGTGAGCC--TTT--AT-----GGGCGCACA

AATCTCGCTGCGGTCGGTGGGCGCCTGACTTACTTGTTTTAGAGTTTGTTGGGTGC-AGC

GCTGGCCGTCGGTGGGGTCAATTAATTGTGTATCACATCCATAATCTATGGTTTAATGTA

GGCCATTTACCTGT

>Ts_muscalingulata_KX095290

CTGAACTTACTCCAGAGTCGAATTGTTCGGTATTGCAATTCAAAGCGGGTGGTAGGCTCC

ATCCAAGGCTAAATATAACACTAGACCGATAGTTAACAAGTACCGTGAGGGAAAGTTGAA

AAGAACTTTGAAGAGAGAGTTAATAGTACGTGAAATCACCCAGAGGTAAACGGGTAAAGC

CGAAATTGGCTGAGAGGCTGATGTCTTCTGGTG-------TCGGTATGCAGTGTGGAGGT

CGATTTGTTTTT--AAAACGTCTCTGTGCTGTTT--TA--GTATCCGCGCTTGTTGGCTG

TTTCCTCTTTGCCTTA-ACCATGATCGACGATGCGGTCGTTTGTAGTGAGAGAAGGTAGC

GATTGAGTCATTTCGGCTCGATTGTATTATAGCTCTCAACTAACT--------TTTG---

-AACAGACTGTGTTGTTGTGCAGTAGCGGTGTGAGCC--TTT---TT----AGGCGCACA

AATTCTGCTGCGGTTGGTGGGCGCTTGACTTACTTGTTTTAGAGTTTGTTGAGTGC-AAC

GCTGGCTGTTGGTGGAGTCA-TTCATTGTGTATCACATCCATAATCTATGGTTTAATGTA

GGCCATTTACCCGT

>Ts_minima_KX095293

CTGAAGTTACTCCAGAGTCGAATTGTTCGGTATTGCAATTCAAAGCGGGTGGTAGGCTCC

ATCCAAGGCTAAATATAACACTAGACCGATAGTTAACAAGTACCGTGAGGGAAAGTTGAA

AAGAACTTTGAAGAGAGAGTTAATAGTACGTGAAATCACCCAGAGGTAAACGGGTAAAGC

CGAAATGGGCTGAGAGGCTGATGTCTTCTGGTGTATTTTCTCGGTATGCGGTGCGGAGGT

CGATTTGTTTT---AGAACGTCTCTGTGTCGTTG--CA--GTACTCGAGCCAGTTGGCTG

TTTCCTCTTAGCCTTA-ACCATGATCGACGATGCGGTCGTTTGTAGTGGAGAAAGGTAGC

GAGTGTGGCATTTCGTCGCGCTTGTGTTATAGTCTCCATGTTCAT--------TGTG---

-AACAGACTGTGTTGTTGTGCAGTGGCGATGTGAGCC--TTC--ATTTG--GGGCGCACA

AATCTCGCTGCGGTCGGTGGGCGCCTGACTTACTTGTTTTAGAGTTTGTTGGGTGC-AGC

GCTGGCCGTCGGTGGGGTCAATTAATTGTGTATCACATCCATAATCTATGGTTTAATGTA

GGCCATTTACCTGT

>Th_alpina_KX095295

CTGAAGTTACTCCAGAGTCGAATTGTTCGGTATTGCAATTCAAAGTGGGTGGTAAACTCC

ATCCAAGGCTAAATATAACACTAGACCGATAGTCGACAAGTACCGTGAGGGAAAGTTGAA

AAGAACTTTGAAGAGAGAGTTAATAGTACGTGAAATCACCCAGAGGTAAACGGGTAAAGC

CGAAATTGGCAGAGAGGCTGATGTCTTCTGGCG--------CAGTATGTGGTTCGGATGT

CGATTTGTTTT---AGAACGACTCTGGACTGT--------GTATTTGTGTTAGTTGGCTG

TTTCCTTTTTGCCTCA-ACCATGATCGACGATGAGGTCATTCATAGTGGTAAAAGGTACC

TATTAAGCCAATT-GGTTTGATAGTGTTATAGCTACCATATCC-------------G---

-AATAGACTTTGTTGTTGTGCAGTTGCGATGTGAGCC--GAAA--------TGGCGCACA

AGTCTTGTTGCGGTTGGTGGGCGCTTGGCTTACTTGTTTTAGAGTCTGCTGAGTGC-ATT

GCTGGCTGTCAGTGAGATAA-----ATGTGTATCACGTCTATAATCCATGGTTTAATGTA

GGCCATTTACCTGT

>Th_capricornia_KX095296

CTGAAGTTACTCCAGAGTCGAATTGTTCGGTATTGCAATTCAAAGTGGGTGGTAAACTCC

ATCCAAGGCTAAATATAACACTAGACCGATAGTCGACAAGTACCGTGAGGGAAAGTTGAA

AAGAACTTTGAAGAGAGAGTTAATAGTACGTGAAATCACCCAGAGGTAAACGGGTAAAGC

CGAAATTGGCAGAGAGGCTGATGTCTTCTGGCG--------CAGTATGTGGTTTGGAGGT

CGATTTGTTTT---AGAACGTCTCTGGGCTGT--------GTATTTGTGTTAGTTGGCTG

TTTCCTTTTTGCTTTA-ACCATGATCGACGATGAGGTCATTCATAGTGGTAAAAGGTATC

TATTAAGCCAATT-GGTTTGATAGTGTTATAGTTACCATATCC-------------G---

-AATAGACTTTGTTGTTGTGCAGTAGCGATGTGAGCC--GTA---------CGGCGCACA

TATCTTGTTGCGGTTGGTGGGCGCTCGGCTTACTTGTTTTAGAGTCTGCTGAGTGC-ATT

GCTGGCTGTCAGCGAGGTCT-----TTGTGTATCACGTCTACAATCCATGGTTTAATGTA

GGCCATTTACCTGT

>Th_comes_KX095297

CTGAAGTTACTCCAGAGTCGAATTGTTCGGTATTGCAATTCAAAGTGGGTGGTAAACTCC

ATCCAAGGCTAAATATAACACTAGACCGATAGTCGACAAGTACCGTGAGGGAAAGTTGAA

AAGAACTTTGAAGAGAGAGTTAATAGTACGTGAAATCACCCAGAGGTAAACGGGTAAAGC

CGAAATTGGTAGAGAGGCTGATGTCTTCTGGCG--------CAGTATGTGGTTCGGAGGT

CGATTTGTTTT---AGAACGTCTCTGGGCTAT--------GTGTTTGTGCTAGTTGGCTG

TCTCCTTTTTGCCTTA-ACCATGATCGACGATGAGGTCATTCACAGTGGTAAAAGGTATC

TGTCAGGCCAATT-GGTCGGATAGTGTTATAGTTACCATATTC-------------G---

-AATAGACTTTGTTGTTGTGCAGTTGCGATGTGAGCC--GTA---------TGGCGCACA

AATCTCGTTGCGGTTGGTGGGTGCTTGGCTTACTTGTTTTAGAGTCTGCTGAGTGC-ATT

GCTGGCCGTCAGTGGGGTAC-----TTGTGTATCACGTCTATAATCCATGGTTTAATGTA

GGCCATTTACCTGT

>Th_comes_KX095298

CTGAAGTTACTCCAGAGTCGAATTGTTCGGTATTGCAATTCAAAGTGGGTGGTAAACTCC

ATCCAAGGCTAAATATAACACTAGACCGATAGTCGACAAGTACCGTGAGGGAAAGTTGAA

AAGAACTTTGAAGAGAGAGTTAATAGTACGTGAAATCACCCAGAGGTAAACGGGTAAAGC

CGAAATTGGCAGAGAGGCTGATGTCTTCTGGCG--------CAGTATGTGGTTCGGAGGT

CGATTTGTTTT---AGAACGTCTCTGGGCTAT--------GTGTTTGTGCTAGTTGGCTG

TCTCCTTTTTGCCTTA-ACCATGATCGACGATGAGGTCATTCACAGTGGTAAAAGGTATC

TATCAGGCCAATT-GGTCGGATAGTGTTATAGTTACCATATTC-------------G---

-AATAGACTTTGTTGTTGTGCAGTTGCGATGTGAGCC--GTA---------TGGCGCACA

AATCTCGTTGCGGTTGGTGGGTGCTTGGCTTACTTGTTTTAGAGTCTGCTGAGTGC-ATT

GCTGGCCGTCAGTGGGGTCC-----TTGTGTATCACGTCTATAATCCATGGTTTAATGTA

GGCCATTTACCTGT

>Th_comes_KX095299

CTGAAGTTACTCCAGAGTCGAATTGTTCGGTATTGCAATTCAAAGTGGGTGGTAAACTCC

ATCCAAGGCTAAATATAACACTAGACCGATAGTCGACAAGTACCGTGAGGGAAAGTTGAA

AAGAACTTTGAAGAGAGAGTTAATAGTACGTGAAATCACCCAGAGGTAAACGGGTAAAGC

CGAAATTGGCGGAGAGGCTGATGTCTTCTGGTG--------CAGTATGTGGTTCGGAGGT

CGATTTGTTTT---AGAACGTCTCTGGGCTAT--------GTGTTTGTGCTAGTTGGCTG

TCTCCTTTTTGCCTTA-ACCATGATCGACGATGAGGTCATTCATAGTGGTAAAAGGTATC

TATCAGGCCAATT-GGTCGGATAGTGTTATAGTTACCATATCC-------------G---

-AATAGACTTTGTTGTTGTGCAGTTGCGATGTGAGCC--GTA---------TGGCGCACA

AATCTCGTTGCGGTTGGTGGGTGCTTGGCTTACTTGTTTTAGAGTCTGCTGAGTGC-ATT

GCTGGCCGTCAGTGGGGTAC-----TTGTGTATCACGTCTATAATCCATGGTTTAATGTA

GGCCATTTACCTGT

>Th_comes_KX095300

CTGAAGTTACTCCAGAGTCGAATTGTTCGGTATTGCAATTCAAAGTGGGTGGTAAACTCC

ATCCAAGGCTAAATATAACACTAGACCGATAGTCGACAAGTACCGTGAGGGAAAGTTGAA

AAGAACTTTGAAGAGAGAGTTAATAGTACGTGAAATCACCCAGAGGTAAACGGGTAAAGC

CGAAATTGGCGGAGAGGCAGATGTCTTCTGGTG--------CAGTATGTGGTTCGGAGGT

CGATTTGTTTT---AGAACGTCTCTGGGCTAT--------GTGTTTGTGCTAGTTGGCTG

TTTCCTTTTTGCCTTA-ACCATGATCGACGATGAGGTCATTCATAGTGGTAAAAGGTATC

TATCAGGCCAATT-GGTCGGTTAGTGTTATAGTTACCATACTC-------------G---

-AATAGACTTTGTTGTTGTGCAGTTGCGATGTGAGCC--GTA---------TGGCGCACA

AATCTCGTTGCGGTTGGTGGGTGCTTGGCTTACTTGTTTTAGAGTCTGCTGAGTGC-ATT

GCTGGCCGTCAGTGGGGTAC-----TTGTGTATCACGTCTATAATCCATGGTTTAATGTA

GGCCATTTACCTGT

>Th_comes_KX095302

CTGAAGTTACTCCAGAGTCGAATTGTTCGGTATTGCAATTCAAAGTGGGTGGTAAACTCC

ATCCAAGGCTAAATATAACACTAGACCGATAGTCGACAAGTACCGTGAGGGAAAGTTGAA

AAGAACTTTGAAGAGAGAGTTAATAGTACGTGAAATCACCCAGAGGTAAACGGGTAAAGC

CGAAATTGGTAGAGAGGCTGATGTCTTCTGGTG--------CAGTATGTGGTTCGGAGGT

CGATTTGTTTT---AGAACGTCTCTGGGCTAT--------GTGTTTGTGCTAGTTGGCTG

TCTCCTTTTTGCCTTA-ACCATGATCGACGATGAGGTCATTCACAGTGGTAAAAGGTATC

TGTCAGGCCAATT-GGTCGGATAGTGTTATAGTTACCATATTC-------------G---

-AATAGACTTTGTTGTTGTGCAGTTGCGATGTGAGCC--GTA---------TGGCGCACA

AATCTCGTTGCGGTTGGTGGGTGCTTGGCTTACTTGTTTTAGAGTCTGCTGAGTGC-ATT

GCTGGCCGTCAGTGGGGTAC-----TTGTGTATCACGTCTATAATCCATGGTTTAATGTA

GGCCATTTACCTGT

>Th_comes_KX095303

CTGAAGTTACTCCAGAGTCGAATTGTTCGGTATTGCAATTCAAAGTGGGTGGTAAACTCC

ATCCAAGGCTAAATATAACACTAGACCGATAGTCGACAAGTACCGTGAGGGAAAGTTGAA

AAGAACTTTGAAGAGAGAGTTAATAGTACGTGAAATCACCCAGAGGTAAACGGGTAAAGC

CGAAATTGGTAGAGAGGCTGATGTCTTCTGGCG--------CAGTATGTGGTTCGGAGGT

CGATTTGTTTT---AGAACGTCTCTGGGCTAT--------GTGTTTGTGCTAGTTGGCTG

TCTCCTTTTTGCCTTA-ACCATGATCGACGATGAGGTCATTCACAGTGGTAAAAGGTATC

TGTCAGGCCAATT-GGTCGGATAGTGTTATAGTTACCATATTC-------------G---

-AATAGACTTTGTTGTTGTGCAGTTGCGATGTGAGCC--GTA---------TGGCGCACA

AATCTCGTTGCGGTTGGTGGGTGCTTGGCTTACTTGTTTTAGAGTCTGCTGAGTGC-ATT

GCTGGCCGTCAGTGGGGTAC-----TTGTGTATCACGTCTATAATCCATGGTTTAATGTA

GGCCATTTACCTGT

>Th_comes_KX095304

CTGAAGTTACTCCAGAGTCGAATTGTTCGGTATTGCAATTCAAAGTGGGTGGTAAACTCC

ATCCAAGGCTAAATATAACACTAGACCGATAGTCGACAAGTACCGTGAGGGAAAGTTGAA

AAGAACTTTGAAGAGAGAGTTAATAGTACGTGAAATCACCCAGAGGTAAACGGGTAAAGC

CGAAATTGGCAGAGAGGCTGATGTCTTCTGGCG--------CAGTATGTGGTTCGGAGCT

CGATTTGTTTT---AGAACGTCTCTGGGCTAT--------GTGTTTGTGCTAGTTGGCTG

TCTCCTTTTTGCCTTA-ACCATGATCGACGATGAGGTCATTCACAGTGGTAAAAGGTATC

TATCAGGCCAATT-GGTCGGATAGTGTTATAGTTACCATATTC-------------G---

-AATAGACTTTGTTGTTGTGCAGTTGCGATGTGAGCC--GTA---------TGGCGCACA

AATCTCGTTGCGGTTGGTGGGTGCTTGGCTTACTTGTTTTAGAGTCTGCTGAGTGC-ATT

GCTGGCCGTCAGTGGGGTCC-----TTGTGTATCACGTCTATAATCCATGGTTTAATGTA

GGCCATTTACCTGT

>Th_comes_KX095305

CTGAAGTTACTCCAGAGTCGAATTGTTCGGTATTGCAATTCAAAGTGGGTGGTAAACTCC

ATCCAAGGCTAAATATAACACTAGACCGATAGTCGACAAGTACCGTGAGGGAAAGTTGAA

AAGAACTTTGAAGAGAGAGTTAATAGTACGTGAAATCACCCAGAGGTAAACGGGTAAAGC

CGAAATTGGCGGAGAGGCTGATGTCTTCTGGCG--------CAGTATGTGGTTCGGAGGT

CGATTTGTTTT---AGAACGTCTCTGGGCTAT--------GTGTTTGTGCTAGTTGGCTG

TTTCCTTTCTGCCTTA-ACCATGATCGACGATGAGGTCATTCATAGTGGTAAAAGGTATC

TATCAGGCCAATT-GGTCGGATAGTGTTATAGTTACCATATCC-------------G---

-AATAGACTTTGTTGTTGTGCAGTTGCGATGTGAGCC--GTA---------TGGCGCACA

AATCTCGTTGCGGTTGGTGGGTGCTTGGCTTACTTGTTTTAGAGTCTGCTGAGTGC-ATT

GCTGGCCGTCAGTGGGGTAC-----TTGTGTATCACGTCTATAATCCATGGTTTAATGTA

GGCCATTTACCTGT

>Th_crotalum_KX095307

CTGAAGTTACTCCAGAGTCGAATTGTTCGGTATTGCAATTCAAAGTGGGTGGTAAACTCC

ATCCAAGGCTAAATATAACACTAGACCGATAGTCGACAAGTACCGTGAGGGAAAGTTGAA

AAGAACTTTGAAGAGAGAGTTAATAGTACGTGAAATCACCCAGAGGTAAACGGGTAAAGC

CGAAATTGGCAGAGAGGCTGATGTCTTCTGGCG--------CAGTATGTGGTTCGGAGGT

CGATTTGTTTT---AGAACGTCTCTGGGCTGT--------GTATTTGTGTTAGTTGGCTG

TTTCCTTTTTGCCTTA-ACCATGATCGACGATGAGGTCATTCATAGTGGTAAAAGGTATC

TATTAAGCCAATT-GGTTTGATAGTGTTATAGTTACCAAATCC-------------G---

-AATAGACTTTGTTGTTGTGCAGTTGCGATGTGAGCC--GTA---------TGGCGCACA

AATCTTGTTGCGGTTGGTGGGCGCTTGGCTTACTTGTTTTAGAGTCTGCTGAGTGC-ATT

GCTGGCTGTCAGTGAGGTAT-----ATGTGTATCACGTCTATAATCCATGGTTCAATGTA

GGCCATTTACCTGT

>Th_crotalum_KX095308

CTGAAGTTACTCCAGAGTCGAATTGTTCGGTATTGCAATTCAAAGTGGGTGGTAAACTCC

ATCCAAGGCTAAATATAACACTAGACCGATAGTCGACAAGTACCGTGAGGGAAAGTTGAA

AAGAACTTTGAAGAGAGAGTTAATAGTACGTGAAATCACCCAGAGGTAAACGGGTAAAGC

CGAAATTGGCAGAGAGGCTGATGTCTTCTGGCG--------CAGTATGTGGTTCGGAGGT

CGATTTGTTTT---AGAACGTCTCTGGGCTGT--------GTATTTGTGTTAGTTGGCTG

TTTCCTTTTTGCCTTA-ACCATGATCGACGATGAGGTCATTCATAGTGGTAAAAGGTATC

TATTAAGCCAATT-GGTTTGATAGTGTTATAGTTACCAGATCC-------------G---

-AATAGACTTTGTTGTTGTGCAGTTGCGATGTGAGCC--GTA---------TGGCGCACA

AATCTTGTTGCGGTTGGTGGGCGCTTGGCTTACTTGTTTTAGAGTCTGCTGAGTGC-ATT

GCTGGCTGTCAGTGAGATAT-----ATGTGTATCACGTCTATAATCCATGGTTTAATGTA

GGCCATTTACCTGT

>Th_munifica_KX095309

CTGAAGTTACTCCAGAGTCGAATTGTTCGGTATTGCAATTCAAAGTGGGTGGTAAACTCC

ATCCAAGGCTAAATATAACACTAGACCGATAGTCGACAAGTACCGTGAGGGAAAGTTGAA

AAGAACTTTGAAGAGAGAGTTAATAGTACGTGAAATCACCCAGAGGTAAACGGGTAAAGC

CGAAATTGGCAGAGAGGCTGATGTCTTCTGGCG--------CAGTATGTGGTTCGGAGGT

CGATTTGTTTT---AGAACGTCTCTGGGCTGT--------GTATTTGTGTTAGTTGGCTG

TTTCCTTTTTGCCTGA-ACCATGATCGACGATGAGGTCATTCATAGTGGTAAAAGGTATC

TATTAAGCCAATT-GGTTTGATAGTGTTATAGTTACCATATCC-------------G---

-AATAGACTTTGTTGTTGTGCAGTTGCGATGTGAGCC--GTA---------TGGCACACA

AATCTTGTTGCGGTTGGTGGGCGCTTGGCTTACTTGTTTTAGAGTCTGCTGAGTGC-ATT

GCTGGCTGTCAGTGAGATAT-----ATGTGTATCACGTCTACAATCCATGGTTTAATGTA

GGCCATTTACCTGT

>Th_pearsoni_KX095310

CTGAAGTTACCTCAGAGTCGAATTGTTCGGTATTGCAATTCAAAGTGGGTGGTAAACTCC

ATCCAAGGCTAAATATAACACTAGACCGATAGTCAACAAGTACCGTGAGGGAAAGTTGAA

AAGAACTTTGAAGAGAGAGTTAATAGTACGTGAAATCACCCAGAGGTAAACGGGTAAAGC

CGAAATTGGCGGAGAGGCTGATGTCTTCTGGCG--------TAGTATGCGGTTTGGAGGT

CGATTCGTTTT---AGAACGTCTCTGGGCTGT--------GTGTTTGTGCTAGTTGGCTG

TTTCCTTTTTGCCTCA-ACCATGATCGACGATGAGGTCATTCGTAGTGGTGGAAGGTATC

TATTAGGCCACTT-GGTTTGATAGTGTTATAGCTGCCATATCCA------------G---

-AATAGACTTTGTTGTTGTGCAGTAGCGATGTGAGCC--GTA---------TGGCGCACA

AATCTTGTTGCGGTTGGTGGGTGCTTGGCTTACTTGTTTTAGAGTTTGCTGAGTGCTGCT

GCTGGCTGTCAGCGGGGTCA-----CTGTGTATCACGTCTATAATCCATGGTTTAATGTA

GGCCATTTACCTGT

>Th_simulator_KX095311

CTGAAGTTACTCCAGAGTCGAATTGTTCGGTATTGCAATTCAAAGTGGGTGGTAAACTCC

ATCCAAGGCTAAATATAACACTAGACCGATAGTCGACAAGTACCGTGAGGGAAAGTTGAA

AAGAACTTTGAAGAGAGAGTTAATAGTACGTGAAATCACCCAGAGGTAAACGGGTAAAGC

CGAAATTGGCAGAGAGGCTGATGTCTTCTGGCG--------TAGTATGTGGTTCAGAGGT

CGATTTGTTTT---AGAACGTCTCTGGGCTAT--------GTGTCTGTGCTAGTTGGCTG

TTTCCTTTTTGCCTTA-ACCATGATCGACGATGAGGTCATTCATAGTGGTAAAAGGTATC

TATTAAGCCAATT-GGTTTGATAGTGTTATAGTTACCATATCC-------------G---

-AATAGACTTTGTTGTTGTGCAGTTGCGATGTGAGCC--GTA---------TGGCGCACA

AATCTTGTTGCGGTTGGTGGGCGCTTGGCTTACTTGTTTTAGAGTCTGCTGAGTGC-ATT

GCTGGCCGTCAGTGGGATAT-----ATGTGTATCACGTCTATAATCCATGGTTTAATGTA

GGCCATTTACCTGT

>Th_simulator_KX095312

CTGAAGTTACTCCAGAGTCGAATTGTTCGGTATTGCAATTCAAAGTGGGTGGTAAACTCC

ATCCAAGGCTAAATATAACACTAGACCGATAGTCGACAAGTACCGTGAGGGAAAGTTGAA

AAGAACTTTGAAGAGAGAGTTAATAGTACGTGAAATCACCCAGAGGTAAACGGGTAAAGC

CGAAATTGGCAGAGAGGCTGATGTCTTCTGGTG--------CAGTATGTGGTTCGGAGGT

CGATTTGTTTT---AGAACGTCTCTGGGCTAT--------GTGTTTGTGCTAGTTGGCTG

TTTCCTTTTTGCCTTA-ACCATGATCGACGATGAGGTCATTCATAGGGGTAAAAGGTATC

TATTAAGCCAATT-GGTTTGATAGTGTTATAGTTACCATATCC-------------G---

-AATAGACTTTGTTGTTGTGCAGTTGCGATGTGAGCC--GTA---------TGGCGCACA

AATCTCGTTGCGGTTGGTGGGCGCTTGGCTTACTTGTTTTAGAGTCTGCTGAGTGC-ATT

GCTGGCTGTCAGTGGGGTAT-----ATGTGTATCACGTCTATAATCCATGGTTTAATGTA

GGCCATTTACCTGT

>Th_simulator_KX095313

CTGAAGTTACTCCAGAGTCGAATTGTTCGGTATTGCAATTCAAAGTGGGTGGTAAACTCC

ATCCAAGGCTAAATATAACACTAGACCGATAGTCGACAAGTACCGTGAGGGAAAGTTGAA

AAGAACTTTGAAGAGAGAGTTAATAGTACGTGAAATCACCCAGAGGTAAACGGGTAAAGC

CGAAATTGGCAGAGAGGCTGATGTCTTCTGGCG--------TAGTATGTGGTTCAGAGGT

CGATTTGTTTT---AGAACGTCTCTGGGCTAT--------GTGTCTGTGCTAGTTGGCTG

TTTCCTTTTTGCCTTA-ACCATGATCGACGATGAGGTCATTCATAGTGGTAAAAGGTATC

TATTAAGCCAATT-GGTTTGATAGTGTTATAGTTACCATATCC-------------G---

-AATAGACTTTGTTGTTGTGCAGTTGCGATGTGAGCC--GTA---------TGGCGCACA

AATCTTGTTGCGGTTGGTGGGCGCTTGGCTTACTTGTTTTAGAGTCTGCTGAGTGC-ATT

GCTGGCCGTCAGTGGGATAT-----ATGTGTATCACGTCTATAATCCATGGTTTAATGTA

GGCCATTTACCTGT

>Th_simulator_KX095314

CTGAAGTTACTCCAGAGTCGAATTGTTCGGTATTGCAATTCAAAGTGGGTGGTAAACTCC

ATCCAAGGCTAAATATAACACTAGACCGATAGTCGACAAGTACCGTGAGGGAAAGTTGAA

AAGAACTTTGAAGAGAGAGTTAATAGTACGTGAAATCACCCAGAGGTAAACGGGTAAAGC

CGAAATTGGCAGAGAGGCTGATGTCTTCTAGCG--------TAGTATGTGGTTCAGAGGT

CGATTTGTTTT---AGAACGTCTCTGGGCTAT--------GTGTCTGTGCTAGTTGGCTG

TTTCCTTTTTGCCTTA-ACCATGATCGACGATGAGGTCATTCATAGTGGTAAAAGGTATC

TATTAAGCCAATT-GGTTTGATAGTGTTATAGTTACCATATCC-------------G---

-AATAGACTTTGTTGTTGTGCAGTTGCGATGTGAGCC--GTA---------TGGCGCACA

AATCTTGTTGCGGTTGGTGGGCGCTTGGCTTACTTGTTTTAGAGTCTGCTGAGTGC-ATT

GCTGGCCGTCAGTGGGATAT-----ATGTGTATCACGTCTATAATCCATGGTTTAATGTA

GGCCATTTACCTGT

>Th_simulator_KX095315

CTGAAGTTACTCCAGAGTCGAATTGTTCGGTATTGCAATTCAAAGTGGGTGGTAAACTCC

ATCCAAGGCTAAATATAACACTAGACCGATAGTCGACAAGTACCGTGAGGGAAAGTTGAA

AAGAACTTTGAAGAGAGAGTTAATAGTACGTGAAATCACCCAGAGGTAAACGGGTAAAGC

CGAAATTGGCAGAGAGGCTGATGTCTTCTGGCG--------TAGTATGTGGTTCAGAGGT

CGATTTGTTTT---AGAACGTCTCTGGGCTAT--------GTGTCTGTGCTAGTTGGCTG

TTTCCTTTTTGCCTTA-ACCATGATCGACGATGAGGTCATTCATAGTGGTAAAAGGTATC

TATTAAGCCAATT-GGTTTGATAGTGTTATAGTTGCCATATCC-------------G---

-AATAGACTTTGTTGTTGTGCAGTTGCGATGTGAGCC--GTA---------TGGCGCACA

AATCTTGTTGCGGTTGGTGGGCGCTTGGCTTACTTGTTTTAGAGTCTGCTGAGTGC-ATT

GCTGGCCGTCAGTGGGATAT-----ATGTGTATCACGTCTATAATCCATGGTTTAATGTA

GGCCATTTACCTGT

>Th_simulator_KX095316

CTGAAATTACTCCAGAGTCGAATTGTTCGGTATTGCAATTCAAAGTGGGTGGTAAACTCC

ATCCAAGGCTAAATATAACACTAGACCGATAGTCGACAAGTACCGTGAGGGAAAGTTGAA

AAGAACTTTGAAGAGAGAGTTAATAGTACGTGAAATCACCCAGAGGTAAACGGGTAAAGC

CGAAATTGGCAGAGAGGCTGATGTCTTCTGGCG--------TAGTATGTGGTTCGGAGGT

CGATTTGTTTT---AGAACGTCTCTGGGCTAT--------GTGTCTGTGCTAGTTGGCTG

TTTCCTTTTTGCCTTA-ACCATGATCGACGATGAGGTCATTCATAGTGGTAAAAGGTATC

TATTAAGCCAATT-GGTTTGATAGTGTTATAGTTACCATATCC-------------G---

-AATAGACTTTGTTGTTGTGCAGTTGCGATGTGAGCC--GTA---------TGGCGCACA

AATCTTGTTGCGGTTGGTGGGCGCTTGGCTTACTTGTTTTAGAGTCTGCTGAGTGC-ATT

GCTGGCTGTCAGTGAGATAT-----ATGTGTATCACGTCTATAATCCATGGTTTAATGTA

GGCCATTTACCTGT

>Th_subulata_KX095317

CTGAAGTTACTCCAGAGTCGAATTGTTCGGTATTGCAATTCAAAGTGGGTGGTAAACTCC

ATCCAAGGCTAAATATAACACTAGACCGATAGTCGACAAGTACCGTGAGGGAAAGTTGAA

AAGAACTTTGAAGAGAGAGTTAATAGTACGTGAAATCACCCAGAGGTAAACGGGTAAAGC

CGAAATTGGCAGAGAGGCTGATGTCTTCTGGCG--------CAGTATGTGGTTCGGAGGT

CGATTTGTTTT---AGAACGTCTCTGGGCTGT--------GTATTTGTGTTAGTTGGCTG

TTTCCTTTTTGCCTTA-ACCATGATCGACGATGAGGTCATTCATAGTGGTAAAAGGTATC

TATTAAGCCAATT-GGTTTGATAGTGTTATAGTTACCATATCC-------------G---

-AATAGACTTTGTTGTTGTGCAGTAGCGATGTGAGCC--GTA---------TGGCGCACA

AATCTTGTTGCGGTTGGTGGGCGCTTGGCTTACTTGTTTTAGAGTCTGCTGAGTGC-ATT

GCCGGCTGTCAGTGAGGTCC-----TTGTGTATCACGTCTACAATCCATGGTTTAATGTA

GGCCATTTACCTGT

>Th_umbella_KX095318

CTGAAGTTACTCCAGAGTCGAATTGTTCGGTATTGCAATTCAAAGTGGGTGGTAAACTCC

ATCCAAGGCTAAATATAACACTAGACCGATAGTCGACAAGTACCGTGAGGGAAAGTTGAA

AAGAACTTTGAAGAGAGAGTTAATAGTACGTGAAATCACCCAGAGGTAAACGGGTAAAGC

CGAAATTGGCGGAGAGGCTGATGTCTTCTGGTG--------CAGTATGTGGTTCGGAGGT

CGATTTGTTTT---AGAACGTCTCTGGGCTGT--------GTATTTGTGTTAGTTGGCTG

TTTCCTTTTTGCCTTA-ACCATGATCGACGATGAGGTCATTCATAGTGGTAAAAGGTATC

TATCAAGCCAATT-GGTTTGATAGTGTTATAGCTACCATATCC-------------G---

-AATAGACTTTGTTGTTGTGCAGTAGCGATGTGAGCC--GTA---------TGGCGCACA

AATCTTGTTGCGGTTGGTGGGCGCTTGGCTTACTTGTTTTAGAGTCTGCTGAGTGC-ATT

GCTGGCTGTCAGTGAGGTCC-----TTGTGTATCACGTCTATAATCCATGGTTTAATGTA

GGCCATTTACCTGT

>Th_verruca_KX095319

CTGAAGTTACTCCAGAGTCGAATTGTTCGGTATTGCAATTCAAAGTGGGTGGTAAACTCC

ATCCAAGGCTAAATATAACACTAGACCGATAGTCGACAAGTACCGTGAGGGAAAGTTGAA

AAGAACTTTGAAGAGAGAGTTAATAGTACGTGAAATCACCCAGAGGTAAACGGGTAAAGC

CGAAATTGGCAGAGAGGCTGATGTCTTCTGGCG--------CGGTATGTGGTTCGGAGGT

CGATTTGTTTT---AGAACGTCTCTGGGCTGC--------GTGTCTGTGCTAGTTGGCTG

TCTCCTTTTTGCTTTA-ACCATGATCGACGATGAGGTCATTCATAGTGGTAAAAGGTATC

TATCAAGCCAATT-GGTTTGTTAGTGTTATAGTTACCATATCC-------------G---

-AATAGACTTTGTTGTTGTGCAGTAGCGATGTGAGCC--GTA---------TGGCGCACA

AATCTTGTTGCGGTTGGTGGGCGCTTGGCTTACTTGTTTTAGAGTCTGCTGAGTGC-ATT

GCTGGCTGTCAGCGGGGTAA-----TTGTGTATCACGTCTATAATCCATGGTTTAATGTA

GGCCATTTACCTGT

>Th_verruca_KX095320

CTGAAGTTACTCCAGAGTCGAATTGTTCGGTATTGCAATTCAAAGTGGGTGGTAAACTCC

ATCCAAGGCTAAATATAACACTAGACCGATAGTCGACAAGTACCGTGAGGGAAAGTTGAA

AAGAACTTTGAAGAGAGAGTTAATAGTACGTGAAATCACCCAGAGGTAAACGGGTAAAGC

CGAAATTGGCAGAGAGGCTGATGTCTTCTGGCG--------CGGTATGTGGTTCGGAGGT

CGATTTGTTTT---AGAACGTCTCTGGGCTGC--------GTGTCTGTGCTAGTTGGCTG

TCTCCTTTTTGCTTTA-ACCATGATCGACGATGAGGTCATTCATAGTGGTAAAAGGTATC

TATCAAGCCAATT-GGTTTGTTAGTGTTATAGTTACCATATCC-------------G---

-AATAGACTTTGTTGTTGTGCAGTAGCGATGTGAGCC--GTA---------TGGCGCACA

AATCTTGTTGCGGTTGGTGGGCGCTTGGCTTACTTGTTTTAGAGTCTGCTGAGTGC-ATT

GCTGGCTGTCAGCGGGGTAC-----TTGTGTATCACGTCTATAATCCATGGTTTAATGTA

GGCCATTTACCTGT

>Th_verruca_KX095321

CTGAAGTTACTCCAGAGTCGAATTGTTCGGTATTGCAATTCAAAGTGGGTGGTAAACTCC

ATCCAAGGCTAAATATAACACTAGACCGATAGTCGACAAGTACCGTGAGGGAAAGTTGAA

AAGAACTTTGAAGAGAGAGTTAATAGTACGTGAAATCACCCAGAGGTAAACGGGTAAAGC

CGAAATTGGCAGAGAGGCTGATGTCTTCTGGCG--------CGGTATGTGGTTCGGAGGT

CGATTTGTTTT---AGAACGTCTCTGGGCTGC--------GTGTCTGTGCTAGTTGGCTG

TCTCCTTTTTGCTTTA-ACCATGATCGACGATGAGGTCATTCATAGTGGTAAAAGGTATC

TATCAAGCCAATT-GGTTTGTTAGTGTTATAGTTACCATATCC-------------G---

-AATAGACTTTGTTGTTGTGCAGTAGCGATGTGAGCC--GTA---------TGGCGCACA

AATCTTGTTGCGGTTGGTGGGCGCTTGGCTTACTTGTTTTAGAGTCTGCTGAGTGC-ATT

GCTGGCTGTCAGCGGGGTAC-----TTGTGTATCACGTCTATAATCCATGGTTTAATGTA

GGCCATTTACCTGT

>Th_verruca_KX095322

CTGAAGTTACTCCAGAGTCGAATTGTTCGGTATTGCAATTCAAAGTGGGTGGTAAACTCC

ATCCAAGGCTAAATATAACACTAGACCGATAGTCGACAAGTACCGTGAGGGAAAGTTGAA

AAGAACTTTGAAGAGAGAGTTAATAGTACGTGAAATCACCCAGAGGTAAACGGGTAAAGC

CGAAATTGGCAGAGAGGCTGATGTCTTCTGGCG--------CGGTATGTGGTTCGGAGGT

CGATTTGTTTT---AGAACGTCTCTGGGCTAT--------GTGTTTGCGCTAGTTGGCTG

TTTCCTTTTTGCTTTA-ACCATGATCGACGATGAGGTCATTCATAGTGGTAAAAGGTATC

TATCAAGCCAATT-GGTTTGTTAGTGTTATAGTTACCATATCC-------------G---

-AATAGACTTTGTTGTTGTGCAGTAGCGATGTGAGCC--GTA---------TGGCGCACA

AATCTTGTTGCGGTTGGTGGGCGCTTGGCTTACTTGTTTTAGAGTCTGCTGAGTGC-ATT

GCTGGCTGTCAGCGGGGTAA-----TTGTGTATCACGTCTATAATCCATGGTTTAATGTA

GGCCATTTACCTGT

>Th_verruca_KX095323

CTGAAGTTACTCCAGAGTCGAATTGTTCGGTATTGCAATTCAAAGTGGGTGGTAAACTCC

ATCCAAGGCTAAATATAACACTAGACCGATAGTCGACAAGTACCGTGAGGGAAAGTTGAA

AAGAACTTTGAAGAGAGAGTTAATAGTACGTGAAATCACCCAGAGGTAAACGGGTAAAGC

CGAAATTGGCAGAGAGGCTGATGTCTTCTGGCG--------CGGTATGTGGTTCGGAGGT

CGATTTGTTTT---AGAACGTCTCTGGGCTAT--------GTGTTTGCGCTAGTTGGCTG

TTTCCTTTTTGCTTTA-ACCATGATCGACGATGAGGTCATTCATAGTGGTAAAAGGTATC

TTTCAAGCCAATT-GGTTTGTTAGTGTTATAGTTACCATATCC-------------G---

-AATAGACTTTGTTGTTGTGCAGTAGCGATGTGAGCC--GTA---------TGGCGCACA

AATCTTGTTGCGGTTGGTGGGCGCTTGGCTTACTTGTTTTAGAGTCTGCTGAGTGC-ATT

GCCGGCTGTCAGCGGGGTAA-----TTGTGTATCACATCTATAATCCATGGTTTAATGTA

GGCCATTTACCTGT

>Th_verruca_KX095325

CTGAAGTTACTCCAGAGTCGAATTGTTCGGTATTGCAATTCAAAGTGGGTGGTAAACTCC

ATCCAAGGCTAAATATAACACTAGACCGATAGTCGACAAGTACCGTGAGGGAAAGTTGAA

AAGAACTTTGAAGAGAGAGTTAATAGTACGTGAAATCACCCAGAGGTAAACGGGTAAAGC

CGAAATTGGCAGAGAGGCTGATGTCTTCTGGCG--------CGATATGTGGTTCGGAGGT

CGATTTGTTTT---AGAACGTCTCTGGGCTAT--------GTGTTTGCGCTAGTTGGCTG

TTTCCTTTTTGCTTTA-ACCATGATCGACGATGAGGTCATTCATAGTGGTAAAAGGTATC

TTTCAAGCCAATT-GGTTTGTTAGTGTTATAGTTACCATATCC-------------G---

-AATAGACTTTGTTGTTGTGCAGTAGCGATGTGAGCC--GTA---------TGGCGCACA

AATCTTGTTGCGGTTGGTGGGCGCTTGGCTTACTTGTTTTAGAGTCTGCTGAGTGC-ATT

GCTGGCTGTCAGCGGGGTAA-----TTGTGTATCACATCTATAATCCATGGTTTAATGTA

GGCCATTTACCTGT

>Didymorchis_sp_AY157163

CTGAGGTTACTCCTGAGTCGAAATGTCTGGTAATGCATTTCAAAGCGGGAGGTAGACTCC

TCCCAAGGCTAAATATAACACTAGTCCGATAGTCGACAAGTACCGTGAGGGAAAGTTGAA

AAGAACTTTGAAGAGAGAGTTAATAGTACGTGAAATCACTCAGAGGTAAACGGGTAAAGC

CGAAATTGGTGGCAAGGCTGATGCCTTCGAGTG--------TGGAATGCCGGGCCAAGGT

CGATTTGTTTT---TGAACGTCTTGGTCTGGT--------GTTTCCGCGCTTGCTGGCTG

TTTCCTTGTTGCCCTAGACCATGATCGACGATTCCGTCGTTTGTACTGGGAGAAGGTAGC

GATCAGA-CATTTTTGTCGGATTGTGTTATAGCTCTCAGCTTTTT---------GCG---

-AACAGATGGAGTTGTTGTGCACTAGCGATGTGAGCG--GACC--TCGGT-CGGCGCACA

AGTATGACTGCGGTTGTCTGGCGTGTGTCTCACTTGTTT-AGAGTTTGGCGCATGT-CTG

GCTGACTGTTGGTTGTGCCT-----CTGTGTATCACGTCAACAATCCATGGTTCAATGTA

GGCCATTTACCTGT
